# Effectiveness of interventions using activity trackers and smartphone applications for increasing physical activity in older adults with health conditions: a systematic review with meta-analysis

**DOI:** 10.1016/j.tjfa.2026.100180

**Published:** 2026-08-31

**Authors:** Nusrat Hamdani, Bruno T Saragiotto, Peter Stubbs, Juliana Oliveira, Anne Tiedemann, Catherine Sherrington, Paloma Santana, Gabriel Ferretti, Arianne Verhagen, Poonam Mehta

**Affiliations:** a Discipline of Physiotherapy, School of Human Performance, Rehabilitation and Population Health, Faculty of Health, University of Technology Sydney, Sydney, Australia; b Women-Newborn-Children’s Service, The Gold Coast University Hospital, Gold Coast, Australia; c Institute for Musculoskeletal Health, School of Public Health, Faculty of Medicine and Health, University of Sydney and Sydney Local Health District, Sydney, Australia; d Masters and Doctoral Programs in Physical Therapy, Universidade Cidade de São Paulo, Sao Paulo, Brazil; e Department of Physical Therapy, Universidade Federal de São Carlos, São Carlos, Brazil

**Keywords:** Aged, Physical activity, Activity trackers, Chronic disease, Rehabilitation

## Abstract

**Background:**

Evidence suggests that physical activity is fundamental to healthy ageing and is associated with prevention of chronic diseases, reduced frailty risk, and premature mortality in older adults.

**Objective:**

To evaluate the effectiveness of interventions using activity trackers and smartphone applications for increasing physical activity (steps per day) in people aged ≥60 years with an underlying health condition.

**Methods:**

Six databases were searched from inception to January 2025. We included randomised controlled trials of interventions that used activity trackers or smartphone applications, to promote physical activity among people aged ≥60 years with an underlying health condition, compared to minimal interventions or other active interventions.

**Results:**

We identified 44 trials (n = 4148). Compared with minimal intervention, the evidence is very uncertain about the effect of activity trackers on physical activity in the short term (near to intervention completion) (MD 1671 steps, 95% CI 1113 to 2229; I² = 90%, 37 trials). At 12 months, activity trackers probably increase physical activity compared with no or minimal intervention (MD 1838 steps, 95% CI 980 to 2696; I² = 64%, 6 trials; moderate certainty). At 6 months and 24 months, the effect of activity trackers on steps was uncertain. Subgroup analyses suggest activity trackers may improve physical activity across metabolic, neurological, and pulmonary conditions based on moderate to low certainty evidence.

**Conclusion:**

Interventions using activity trackers may increase physical activity in the short term, although the evidence is very uncertain, and probably increase physical activity at approximately 12 months. Effects at intermediate-term and 24-month follow-up remain uncertain. Effects on mobility, quality of life and mental health were unclear or small.

**Registration:**

CRD42024622697

## 1 Introduction

Physical activity is critical for healthy ageing [1,2]. Evidence supports the role of regular physical activity in both the primary and secondary prevention of numerous chronic diseases, including cardiovascular disease [3,4], type 2 diabetes [[5], [6], [7]], various forms of cancer [[8], [9], [10]], hypertension [[11], [12], [13]], obesity [14], depression [[15], [16], [17]], and musculoskeletal conditions [18,19]. Moreover, consistent engagement in physical activity is associated with a significant reduction in premature mortality [18,20] and reduces the risk of frailty [21].

The World Health Organization has endorsed the Global Action Plan on Physical Activity (GAPPA) 2018–2030 [22] aiming for a 15% relative decrease in insufficient physical activity by 2030. GAPPA includes 20 policy steps to provide equitable opportunities and promote physical activity, as well as technology-assisted interventions, including activity trackers such as smartphone applications and wearable devices (e.g., smartwatches, smart bands). Activity trackers are wearable, non-invasive devices that monitor physical activity parameters such as steps, energy expenditure, sleep duration, and activity intensity [23,24]. When integrated into comprehensive programs, activity trackers may improve physical activity engagement, quality of life, stress management, sedentary behaviour reduction, and weight management [25,26]. Regular physical activity is a key determinant of healthy ageing, with evidence from longitudinal cohorts [27] and interventional trials [28] demonstrating improvements in physical health and overall ageing outcomes among older adults who are active.

Activity trackers can motivate people and increase daily physical activity [[29], [30], [31], [32], [33], [34]]. While systematic reviews have examined activity trackers in older adults [1,[35], [36], [37], [38], [39]] and on mixed-age populations [29,30,40] most included individuals <60 years, healthy populations, or mixed populations (healthy and diseased) limiting the generalisability to people with underlying health conditions [1,29,30,35,[40], [41], [42], [43], [44], [45]]. Previous reviews have methodological limitations, such as including only community based populations [1] or limiting to usual care comparators [30,35,37,45,46], English-language restriction [37,[41], [42], [43],^46^,^47^], inclusion of traditional pedometers/accelerometers but not modern multifunctional devices or apps [30,37,[46], [47], [48]], non-randomised controlled trial (RCT) designs [1,30], and absence of Grading of Recommendations Assessment, Development and Evaluation (GRADE) assessment [30,41,43,46,47].

This systematic review aimed to summarise the effectiveness of using activity trackers and smartphone applications (apps) on physical activity in adults aged 60 years and older with underlying health conditions. The secondary aim was to investigate the effect on additional outcomes of mobility, quality of life and mental health.

## 2 Methods

The Preferred Reporting Items for Systematic Reviews and Meta-Analyses (PRISMA) guidelines [49] was used to guide appropriate reporting. The protocol was pre-registered in the PROSPERO database (CRD42024622697).

### 2.1 Eligibility criteria

#### 2.1.1 Type of study design

We included randomised controlled trials (RCTs).

#### 2.1.2 Type of participants

We included trials with older adults (≥60 years) that had a pre-existing diagnosed chronic health condition and were recruited from the community, primary health centres, hospital settings, outpatient hospital clinic, rehabilitation setting and general practitioner clinic and day care centres. Studies were eligible if they included participants with a range of pre-existing health conditions such as chronic pain, cardiovascular disorders (e.g., coronary heart disease), respiratory conditions (e.g., COPD), metabolic disorders (e.g., diabetes mellitus), musculoskeletal conditions (e.g., osteoarthritis), cancer, neurological conditions (e.g., stroke), and chronic conditions (including mixed groups of individuals with metabolic, cardiopulmonary, neurological, cardiovascular, musculoskeletal conditions, and frailty). Participants of interest were older adults able to walk with or without an assistive device, as our primary outcome was step count physical activity. Trials that included participants with general good health without any major chronic conditions (metabolic such as diabetes, pulmonary such as COPD, neurological such as stroke, cardiovascular such as ischemic heart disease, musculoskeletal such as arthritis- conditions and frailty) were excluded from the study.

#### 2.1.3 Type of intervention

We included studies evaluating the effectiveness of activity trackers to increase physical activity, as standalone interventions, combined with physical activity counselling, or incorporated as a key component of a broader physical activity interventions (including remote monitoring via tablet or telerehabilitation). Eligible activity trackers included wearable devices (i.e., pedometers) or smartphone applications capable of tracking physical activity measures alone or with a wearable device.

#### 2.1.4 Type of comparators

We compared activity trackers with a) no or minimal interventions, including waiting list or usual care, or b) other active interventions (e.g., exercise prescription etc.). The term ‘usual care’ is often used inconsistently across studies, varying from no intervention to conventional or standard clinical practice. In this review, we relied on each study’s definition of usual care and extracted relevant details on what it entails. If the description was unclear or insufficient, we attempted to contact the study authors for clarification.

#### 2.1.5 Type of outcomes

The primary outcome of interest was physical activity measured as steps per day recorded by an activity tracker (i.e., pedometer, accelerometer, or smartphone-based application).

The secondary outcomes were (ⅰ) mobility, including any validated method measuring walking tests (e.g., 6 Minute walk test - 6MWT, 2 Minute walk test -2MWT), sit to stand, functional tests (e.g., Timed Up and Go-TUG) or other mobility scales; (ⅱ) quality of life, including any validated questionnaire of health-related quality of life, health status or functional status (e.g., Euro-Quality of Life 5-Dimension 5-Level- Euro-QoL 5D-5 L, Short Form survey-36-general health); and (ⅲ) mental health, including self - reported validated questionnaires of depression and anxiety [Short Form survey-36: mental health component, and Hospital Anxiety and Depression Score (HADS)].

Timepoints were categorised as i) short-term: data point closest to the end of the intervention, ii) intermediate-term: data point closest to six months post-intervention but less than 12 months, iii) long-term: data point closest to 12 months post-intervention, and iv) very long-term: data point closest to 24 months post-intervention. The primary endpoint was short-term (post-intervention-data point closest to the end of the intervention period).

### 2.2 Information sources

A systematic literature search was conducted in MEDLINE (Ovid), Embase (Ovid), PsycINFO (Ovid), the Physiotherapy Evidence Database (PEDro), SPORT Discus (EBSCO), and CINAHL (EBSCO). Searches were conducted from inception to January 2025. Reference lists of relevant reviews and trials were also screened. No language restrictions were applied. The full search strategies for each database are provided in the **Appendix**.

### 2.3 Selection process

**Fig. 1:**
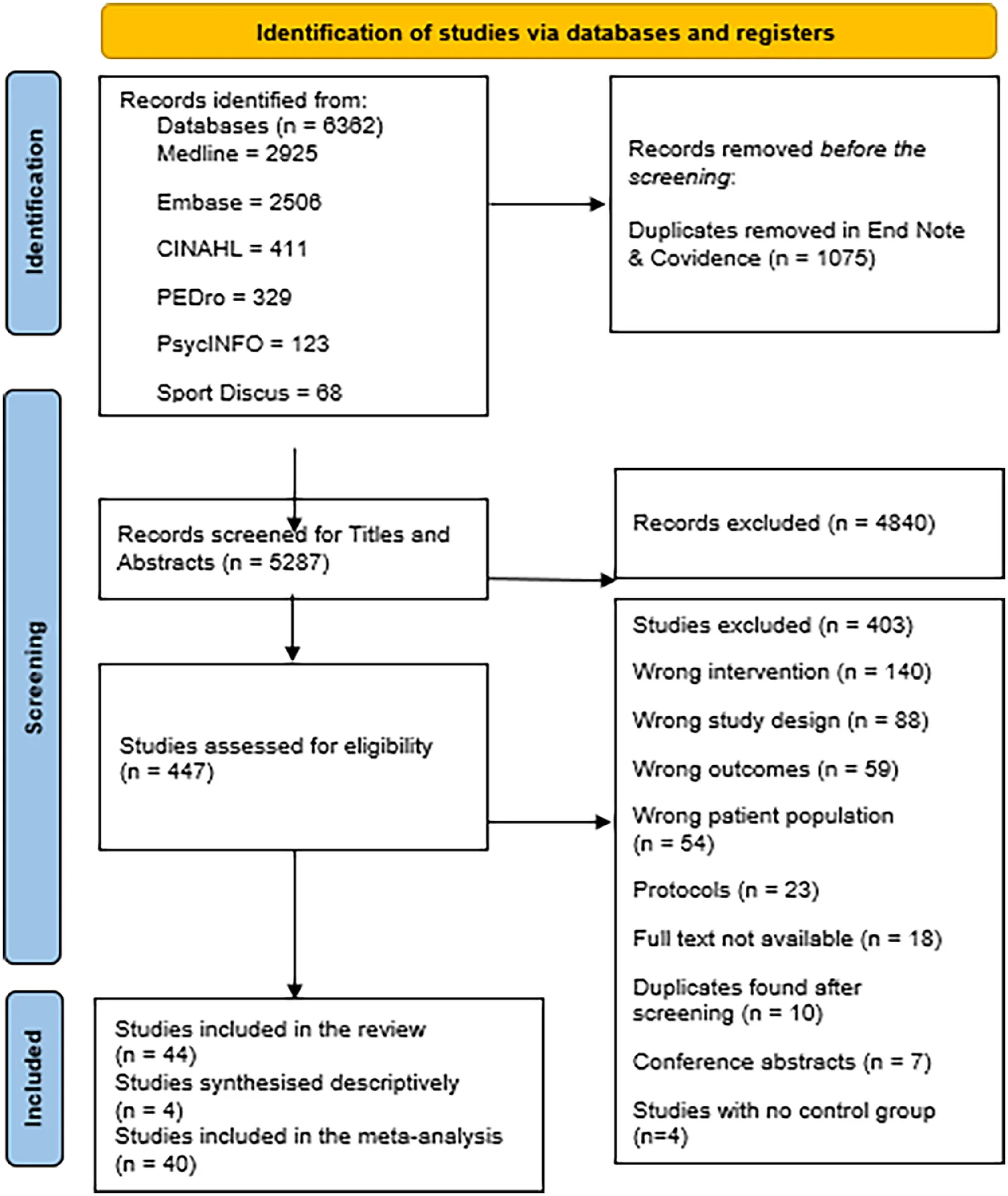
Flowchart of study inclusion through the review.

Following the search, duplicates were removed using Endnote 21 before importing into Covidence (www.covidence.org; Veritas Health Innovation). Titles and abstracts were independently screened by a pair of review authors (NH/PS/PM/PSt). At each step, if a decision regarding eligibility differed among any pair, a third reviewer (BTS) consulted with each pair to reach consensus. NH and PS independently assessed full-text articles for eligibility. Discrepancies were resolved through discussion, with a third author (BTS) consulted if consensus was not reached. Reasons for exclusion were documented in the PRISMA flow diagram (Fig. 1).

Two out of three independent reviewers (NH, PS or GMF) extracted data on the following variables: author, year of publication, country, sample characteristics (e.g., sample size, age, sex, setting), funding and intervention details (e.g., duration, type of intervention, type of device). Duplicate data was compared for accuracy by a third reviewer (BTS).

Outcome data included baseline and follow-up scores for physical activity levels, mobility, quality of life (QOL), and mental health. Post-intervention scores or change scores were used for analysis and change scores were preferred if studies presented both.

When multiple mobility outcomes were reported, we preferentially extracted data in the following order: walking tests (6MWT), functional mobility measures (TUG), and performance-based tests (de Morton Mobility Index). For multiple measures of quality of life, we prioritised health-related quality of life health status (Euro-QoL 5D-5 L), and functional status (Short Form Health Survey 36 - General health). In studies that reported multiple measures of mental health, we selected the outcomes in the following hierarchical order: depression score (Geriatric Depression Scale-15 score), Hospital Depression Score, and Short Form-36 - Emotional well-being. For all the outcomes included in this study please refer to the table in supplementary file 1.

If standard deviations were not available, we calculated these from standard errors or confidence intervals (CIs), using the Revman calculator [50,51]. The intention-to-treat approach was preferred over as-treated or per-protocol.

### 2.4 Methodological quality assessment

We assessed the methodological quality of the included studies using the PEDro scale [52]. The PEDro Scale ranges from 0 (very low methodological quality) to 10 (high methodological quality). Scores and ratings were retrieved from the PEDro database (https://www.pedro.org.au/) and cross-checked by one author (NH). Studies not indexed on PEDro were assessed by two trained PEDro raters (NH and BTS) and discrepancies were resolved by discussion between reviewers. We defined high-quality studies as those with PEDro scores ≥6 and low-quality studies with PEDro scores <6 [53].

### 2.5 Effect measures

We calculated mean differences (MD) with 95% confidence intervals (CI) when studies used continuous outcome variables on the same measurement scale [51]. When different scales per outcome were used, we used standardised mean differences (SMD) with 95% CIs to account for variability across studies and provide a standard metric for comparison [51]. For steps per day, we defined a clinically important difference between groups as 775 steps per day [54]. Effect sizes from SMDs using Cohen were categorised as trivial (SMD < 0.2), small (0.2 to 0.4), medium (0.5 to 0.8), and large effect (SMD >0.8) [55]. We have defined a clinically important difference as 0.3 [56]. Meta-analyses were conducted using a random-effects model in the Review Manager Web [50]. Statistical heterogeneity was assessed using I² statistic, where values below 30% were considered low, 30–60% moderate, 50–90% substantial, and 75–100% considerable heterogeneity [51].

#### 2.5.1 Sensitivity analysis

We conducted sensitivity analysis to assess the robustness of the meta-analysis results. We repeated the analysis by excluding the studies of low quality using random effects model.

#### 2.5.2 Exploratory subgroup analysis

Participants were stratified post hoc into subgroups based on clinical conditions represented in the sample: cancer, chronic conditions, respiratory conditions- chronic obstructive pulmonary disease (COPD), cardiovascular diseases (CVD), metabolic disorder- diabetes mellitus (DM), musculoskeletal conditions (MSK), and neurological conditions- stroke.

### 2.6 Certainty of evidence

We assessed the certainty of evidence for each outcome using GRADE as recommended by the Cochrane Handbook [51]. We interpreted results from the certainty of evidence in the results section using the GRADE guidelines 26 [57].

We used the following criteria to downgrade the certainty of evidence based on the five GRADE considerations:

Study limitations: downgrade by one level if 25% or more of the participants in the comparison were from studies with low methodological quality (defined as a PEDro score of <6) and downgrade by two levels if this proportion reached 50% or over. We also considered whether the methodological quality influences the direction or magnitude of the effect. We used sensitivity analyses (for comparisons with more than 10 studies) to explore this impact i.e., by assessing whether removing studies with low methodological quality alters the effect estimate. If the effect remained stable, we did not downgrade.

Inconsistency: downgrade by one level if I^2^ test >50%; downgrade by 2 levels if I^2^ >75%. For meta-analyses with fewer than 10 studies, we relied on visual inspection and the range of effect sizes to assess inconsistency, rather than I², as the test may be unreliable with a small number of studies.

Indirectness: downgrade by one level if more than 50% of participants vary from the population of interest (e.g., mixed populations or multiple disorders).

Imprecision: downgrade by one level if, in continuous outcomes, the total sample size was < 400 participants; or when the 95% confidence interval around the pooled or best estimate of effect included no effect and the confidence interval crossed an effect size of SMD: 0.5 or MD >10% of the scale in either direction; downgraded the evidence by two levels when there is imprecision due to both of the above.

Publication bias: downgrade by one level if publication bias was identified. If there are more than 10 trials in the analysis, we assessed publication bias by visually inspecting the funnel plots for asymmetry. To statistically evaluate asymmetry, Egger’s test was performed, regressing the MD on the inverse of its SE using an ordinary least squares (OLS) model. A statistically significant intercept (p < 0.05) suggests small-study effects or publication bias. We analysed using Python 3.10 in Google Collab, utilising the stats models package for Egger’s test and matplotlib for visualisation [58].

### 2.7 Deviations from the protocol

We included a post hoc subgroup analyses based on clinical conditions to enhance interpretability and adding sensitivity analyses to address heterogeneity.

## 3 Results

After screening 6362 records, 44 studies were included in the review, of which 40 (participants, n = 4020 contributed data to the meta‑analysis (Fig. 1). Four studies (participants, n = 128) were synthesised narratively due to differences in control groups. Trials were published between 2003 and 2025, conducted across 15 countries, most being from high‑income countries with only two from upper middle income [59,60] and one study conducted in a lower-middle income country [61], and none from low-income countries [62].

### 3.1 Participants

The mean age of participants ranged from 60 years to 81 years, and all included studies recruited both men and women except one study that only included women (breast cancer survivors) [63]. Studies included a range of pre-existing chronic health conditions, such as chronic pain [64], cardiovascular disorders [[65], [66], [67], [68], [69]], respiratory conditions (COPD) [[70], [71], [72], [73], [74], [75], [76], [77]], metabolic disorders (diabetes mellitus) [[78], [79], [80], [81], [82], [83], [84]], musculoskeletal conditions (osteoarthritis) [59,85,86], cancer [63,87,88], neurological conditions (stroke) [[89], [90], [91]], and chronic conditions (including mixed groups of individuals with metabolic, cardiopulmonary, neurological, cardiovascular, musculoskeletal conditions, and frailty) [64,[92], [93], [94], [95], [96], [97]]. Recruitment settings varied including community, primary care, hospital, and rehabilitation settings.

### 3.2 Intervention

Fourteen trials [61,63,64,66,69,74,75,77,79,80,86,90,91,96] reported activity tracker as the primary intervention. The remaining trials used activity trackers as a part of an intervention program. Intervention duration ranged from 1 to 65 weeks (median of 12 weeks). Four studies [59], [60], [64], [92] used activity trackers alongwith a compatible app. as part of physical activity intervention. Three studies [[66], [78], [98]] used smartphone app to increase physical activity along with an activity tracker to count steps (see supplementary file 1).

### 3.3 Comparators

Forty trials compared activity trackers with minimal intervention (no intervention [80,83], usual care [66,68,71,75,77,84,85,87,[89], [90], [91], [92],^99^,^100^], advice [60,65,69,70,[72], [73], [74],^76^,^78^,^79^,^81^,^82^,^86^,[93], [94], [95], [96]], waitlist control [59,64,67,88], delayed intervention [63] or attention control [97]); and four compared activity trackers with other active interventions (activity trackers [101], exercise prescription [61,98] or resistance training [102] (see supplementary file 1).

### 3.4 Outcome measures

For physical activity measurement, a variety of activity trackers were used, including pedometers [61,65,[67], [68], [69], [70],[74], [75], [76], [77], [78], [79], [80], [81],[83], [84], [85], [86],^94^,^95^,^98^,^100^] accelerometers [71,72,[87], [88], [89],^92^,^96^,^102^], and multifunctional wearables (Fitbit [59,64,66,73,90,91,93,99,101], smart sports bracelet [60], Garmin [63,90]), and two trials did not specify device type [82,97].

For mobility, trials reported the 6MWT [70,92,98,99,101], Sit to Stand [88],Timed up and Go (TUG) [94,95], 2MWT [100], Chair Stand test [86], and de Morton mobility index [90]. For quality of life, trials reported the Health-related quality of life CAT (chronic obstructive pulmonary disease (COPD) assessment test) [70,72,73], Short Form (SF)-36 physical health score [92], Short Form (SF)-12 [99], EQ5D-5 L [69,94,96,98,100], EuroQol Visual Analogue Scale (EQ-VAS score) [101], 8-item Short-Form Survey-Physical Component Summary [95,102], Physical-health–related Quality of life [67], Health-Related Quality of Life (HRQOL) by MacNew Heart Disease [68], European Organisation for the Research and Treatment of Cancer Quality of Life Core Questionnaire (EORTCQLQ-C30) [103], St. George's respiratory questionnaire (SGRQ) [71,74], and Chronic respiratory questionnaire (CRQ) [75]. Finally, for mental health they reported Hospital Anxiety and Depression (HADs) questionnaire [70,100], Short Form (SF)-36 (mental health score) [92,99], Short Form 6 dimensions questionnaire (mental health domain) [90], 8-item Short-Form Survey- Mental Component Summary [95,102], 9-item Patient Health Questionnaire [67], 21-item Depression Anxiety Stress Scale 21 (DASS-21) [68], and 9 depression scale (PHQ-9) [103].

Details of all included trials, including participants, interventions, comparison groups and outcomes, are summarised in Supplementary file 1.

### 3.5 Methodological quality

The total PEDro Score ranged from 4 to 8 (median=6) (Table 1). Thirty-two trials were considered of high methodological quality and 12 were of low quality. Overall, sixteen trials [60,61,63,65,73,76,81,[86], [87], [88], [89],^91^,^94^,^98^,^100^,^103^] did not use an intention-to-treat analysis, just one trial included blinded therapists [101], and only two trials blinded subjects [63,69]. Sixteen trials included blinded assessors [59,67,68,74,75,77,82,84,88,90,91,[94], [95], [96], [97],^101^].

**Table 1:** Methodological Quality of the Included Studies According to the PEDro Scale.

	Study	Pedro scale item^a^											Pedro score
		1^b^	2	3	4	5	6	7	8	9	10	11	(0 to 10)
1.	Alonso 2019	Y	Y	N	Y	N	N	N	Y	Y	Y	Y	6
2.	Arbillaga 2018	Y	Y	N	Y	N	N	N	N	Y	Y	Y	5
3.	Ashizawa 2022	Y	Y	N	Y	N	N	N	Y	N	Y	Y	5
4.	Bade 2021	Y	Y	N	Y	N	N	N	Y	N	Y	Y	5
5.	Brickwood 2021	Y	Y	Y	Y	N	N	N	N	Y	Y	Y	6
6.	Bronas 2024	Y	Y	N	Y	N	N	N	Y	Y	Y	Y	6
7.	Chudowolska 2020	Y	Y	Y	Y	N	N	N	N	N	Y	Y	5
8.	Cruz 2016	Y	Y	Y	Y	N	N	N	N	Y	Y	Y	6
9.	Dasgupta 2017	Y	Y	N	Y	N	N	N	N	Y	Y	Y	5
10.	De Greef 2011	Y	Y	Y	Y	N	N	N	Y	Y	Y	Y	7
11.	Demeyer 2017	N	Y	Y	Y	N	N	N	Y	Y	Y	Y	7
12.	De Oliveira 2024	Y	Y	N	Y	N	N	N	N	N	Y	Y	4
13.	Dodson 2025	Y	Y	N	Y	N	N	N	Y	Y	Y	Y	6
14.	Dohrn 2017	Y	Y	Y	Y	N	N	N	N	Y	Y	Y	6
15.	Duscha 2018	Y	Y	N	Y	N	N	N	Y	Y	Y	Y	6
16.	Fanning 2022	Y	Y	Y	Y	N	N	N	Y	Y	Y	Y	7
17.	Gell 2024	Y	Y	N	Y	N	N	Y	Y	N	Y	Y	6
18.	Grau 2020	Y	Y	N	Y	N	N	N	N	N	Y	Y	4
19.	Hassett 2020	Y	Y	Y	Y	N	N	Y	Y	Y	Y	Y	8
20.	Heron 2017	Y	Y	Y	Y	N	N	N	Y	N	Y	Y	6
21.	Hill 2024	Y	Y	Y	Y	N	N	Y	N	N	Y	Y	6
22.	Hinrichs 2016	Y	Y	Y	Y	N	N	Y	N	Y	Y	Y	7
23.	Horniks 2015	Y	Y	N	Y	N	N	N	Y	N	Y	Y	5
24.	Kanai 2018	Y	Y	Y	Y	N	N	Y	Y	N	Y	Y	7
25.	Kenfield 2019	Y	Y	Y	Y	N	N	N	Y	N	Y	Y	6
26.	Kirk 2009	Y	Y	Y	Y	N	N	Y	Y	Y	Y	Y	8
27.	Kitamura 2024	Y	Y	N	Y	N	N	Y	N	Y	Y	Y	6
28.	Li 2020	Y	Y	Y	Y	N	N	Y	Y	Y	Y	Y	8
29.	Li 2023	Y	Y	N	Y	N	N	N	Y	N	Y	Y	6
30.	Lynch 2019	Y	Y	Y	Y	Y	N	N	Y	N	Y	Y	7
31.	McMahon 2024	Y	Y	Y	Y	N	N	Y	Y	Y	Y	Y	8
32.	Mendoza 2015	Y	Y	N	Y	N	N	Y	Y	Y	Y	Y	7
33.	Menkin 2019	Y	Y	Y	Y	N	N	Y	Y	Y	Y	Y	8
34.	Nagai 2018	Y	Y	N	Y	N	N	N	N	Y	Y	Y	5
35.	Nolan 2017	Y	Y	N	Y	N	N	Y	N	Y	Y	Y	6
36.	Smith 2024	Y	Y	Y	N	N	Y	Y	Y	Y	Y	Y	8
37.	Su 2024	Y	Y	Y	Y	N	N	Y	N	Y	Y	Y	7
38.	Talbot 2003	Y	Y	N	Y	N	N	N	Y	N	Y	Y	5
39.	Tew 2015	Y	Y	Y	N	Y	N	N	Y	Y	Y	Y	7
40.	Varas 2018	Y	Y	N	Y	N	N	N	N	N	Y	Y	4
41.	Van 2013	Y	Y	N	Y	N	N	N	Y	Y	Y	Y	6
42.	Widyastuti 2018	Y	Y	N	Y	N	N	N	Y	N	Y	Y	5
43.	Wootton 2019	Y	Y	Y	Y	N	N	Y	N	Y	Y	Y	7
44.	Yates 2009	Y	Y	Y	Y	N	N	Y	Y	Y	Y	Y	8

Abbreviations: Y = yes, N = no.

a1 = Eligibility criteria and source of participants, 2 = random allocation, 3 = concealed allocation, 4 = baseline comparability, 5 = blinded participants, 6 = blinded therapists, 7 = blinded assessors, 8 = adequate follow-up, 9 = intention-to-treat analysis, 10 = between-group comparisons, 11 = point estimates and variability.

Item 1b does not contribute to the total score.

### 3.6 Effects of activity trackers compared to no or minimal interventions

#### 3.6.1 Physical activity

Thirty-seven trials assessed physical activity using a step count, of which 29 (78%) were rated as high methodological quality.

Activity‑tracker interventions were associated with higher daily step count in the short term compared to no or minimal intervention (MD 1671 steps/day, 95% CI 1113 to 2229; I² = 90%, 37 trials, n = 3670; see supplementary file 2, Figure 2a). The certainty of evidence was very low (downgraded one level for study limitations and two levels for inconsistency), so we are very uncertain about the size of this effect (see Supplementary file 2, Table 2 & 3). The sensitivity analysis restricted to high quality trials (29 trials) showed that activity trackers were associated with higher step counts in the short term (MD 1497 steps/day, 95% CI 1040 to 1955; I² = 81%, n = 2698; see supplementary file 2, Figure 2b). The certainty of this sensitivity analysis was low (downgraded two levels for inconsistency), indicating that activity trackers may increase steps in the short term, although heterogeneity remains.

Three, all high-quality trials, reported intermediate-term follow-up. The pooled estimate was imprecise (MD 1048 steps/day, 95% CI −963 to 3058; I² = 77%, n = 263; see supplementary file 2, Figure 2a). Given that the 95% CIs range from both a reduction and a substantial increase in steps, and the certainty of evidence was very low (downgraded two levels for inconsistency and one level for imprecision), the effect at intermediate follow‑up is very uncertain (see Supplementary file 2, Tables 2 and 3).

Six trials reported one‑year follow-up. Activity trackers probably increase daily step count at around 12 months compared with no or minimal intervention (MD 1838 steps/day, 95% CI 980 to 2696, I^2^=64%, n = 643; see Supplementary file 2, Figure 2a), with moderate certainty evidence (downgraded one level for inconsistency) (see supplementary file 2, Table 2&3). The sensitivity analysis of five high‑quality studies was consistent with this finding (MD 1730 steps/day, 95% CI 782 to 2679; I² = 67%, 5 trials, n = 610; moderate certainty; see supplementary file 2, Figure 2b)

A single high-quality study reported two-year follow-up. The effect was imprecise (MD 315 steps/day, 95% CI −1166 to 1796; n = 53; see supplementary file 2, Figure 2a). Because the confidence interval includes both fewer steps and potentially worthwhile increases, and the certainty was low (downgraded two levels for imprecision), the very long-term effect is uncertain (see Supplementary file 2, Tables 2 and 3)

A subgroup analysis was conducted by stratifying the participants according to their clinical condition [see supplementary file 3, Figures 6 (a)]. We found increases in steps/day for diabetes mellitus, stroke, COPD, cancer, and chronic conditions, with no clear effect for musculoskeletal or cardiovascular conditions. Full subgroup estimates are provided in supplementary file 3, Table 5. For the summary of the certainty of evidence (GRADE) see supplementary file 3, Table 4.

A sensitivity analysis (based on high quality studies only) was conducted, which reported a reduced overall average of 1497 steps per day (95% CI 1040 to 1955, I^2^ = 81%, n = 2698) with low certainty of evidence (downgraded two levels for inconsistency). The analysis reported increases in steps/day for cancer, chronic conditions, COPD, cardiovascular diseases, and diabetes mellitus, with no clear benefit for musculoskeletal conditions and stroke (Figures 6 (b), see supplementary file 3).

#### 3.6.2 Mobility

The evidence for the effect of activity trackers and apps on mobility in the short-term is inconclusive (SMD:0.08, 95% CI - 0.27 to 0.42, I^2^=86%, 9 trials, n = 1367) with very low certainty of evidence (downgraded two levels for inconsistency and one level for imprecision) (see supplementary file 2,Figure 4 (a), Table 2 & 3). No trials measured mobility at intermediate, long-term, or very long-term follow ups.

#### 3.6.3 Quality of life

Activity trackers may result in little to no difference in quality of life in the short-term (SMD: ࢤ0.03,95% CI −0.43 to 0.37, I^2^=94%, 16 trials, n = 1960). The certainty of evidence was low (downgraded two levels for inconsistency) (see supplementary file 2, Figure 4 (a), Table 2 & 3). No trials measured quality of life at intermediate, long-term, or very long-term follow ups.

#### 3.6.4 Mental health

Activity trackers result in little to no difference in mental health in the short-term (SMD: 0.07, 95% CI − 0.09 to 0.23, I^2^=49%, 9 trials, n = 1578). The certainty of evidence was high (see supplementary file 2, Figure 4 (a), Table 2 & 3). No trials reported intermediate, long-term, or very long-term follow up.

#### 3.6.5 Exploratory subgroup analyses for secondary outcomes

Two trials reported benefits for mental health in chronic disease conditions (SMD = 0.26, 95% CI 0.03 to 0.49) The certainty of evidence was moderate (downgraded one level for imprecision) (see supplementary file 3, Figure 7 (a) to Figure 9 (b), Table 4 &5).

### 3.7 Effects of activity trackers compared to other active interventions

Meta‑analysis was not undertaken due to differences in control group conditions across studies; results were therefore synthesised narratively; certainty of evidence was assessed using GRADE.

#### 3.7.1 Physical activity

In this comparison three studies reported short‑term outcomes; however, the results were not pooled. In one study [102], the intervention group reported a higher mean value than the control group, with a mean difference of 219 (95% CI −733 to 1171). In another study [101], the mean difference between groups was 341 (95% CI −2214.3 to 2896), favouring the intervention group. While in a third study [61], the mean difference between groups was −92 (95% CI −1710.33 to 1526.33), favouring the control group. For all the studies, the confidence intervals included the null value (see supplementary file 2, Figure 3). The certainty of evidence was very low, (downgraded two levels for study limitations and two levels for imprecision), and the CIs is compatible with both fewer steps and a clinically worthwhile increase, so the true effect remains uncertain (see supplementary file 2, Table 2 & 3). No intermediate, long-term, or very long-term follow-up data were available for this comparison.

#### 3.7.2 Mobility

Two studies reported mobility outcomes. In one study, the intervention group demonstrated lower mean values than the control group, with a standardized mean difference (SMD) of −0.94 (95% CI −1.67 to −0.21) [98]. In another study, the SMD was − 0.10 (95% CI −1.03 to 0.83), favouring the intervention group [101] . When considered together, the confidence intervals across studies included both positive and negative values (see supplementary file 2, Figure 5). The certainty of the evidence was very low (downgrade one level for study limitations and two levels for imprecision) (see supplementary file 2, Table 2 & 3). No trials reported mobility at intermediate, long-term, or very long-term follow ups.

#### 3.7.3 Quality of life

Three studies reported quality‑of‑life outcomes. In one study, the intervention group showed higher mean values than the control group, with an SMD of ... 3.35 (95% CI − 4.45 to − 2.26) [98]. In another study, the SMD was 0.00 (95% CI −0.61 to 0.61) [102], while in a third study, the SMD was 0.97 (95% CI −0.02 to 1.97) [101]. Across studies, confidence intervals included the null value (see supplementary file 2, Figure 5). The certainty of the evidence was very low (downgraded one level for study limitations, two levels for inconsistency and two levels for imprecision) (see supplementary file 2, Table 2 & 3). No trials measured quality of life at intermediate, long-term, or very long-term follow ups.

#### 3.7.4 Mental health

One study [102] reported mental health outcomes with an effect estimate between the intervention and control groups SMD −0.08 (95% CI −0.70 to 0.54) (see supplementary file 2, Figure 5). The certainty of the evidence was very low (downgrade by one level for study limitations and two levels for imprecision) (see supplementary file 2, Table 2 & 3). No trials reported intermediate and long-term follow up.

#### 3.7.5 Adverse events

Six trials [59,70,72,75,86,99] reported adverse events. Intervention-related events included general malaise, fatigue, dizziness, fainting, dyspnoea, chest discomfort, palpitations, musculoskeletal injuries (joint pain, muscle pain, falls, fractures, sprains, back pain), and infections (cold, flu, pneumonia). Non-intervention-related events were reported in three studies [68,88,103]; including minor musculoskeletal strains, similar unanticipated events across both groups (musculoskeletal issues, flu-like symptoms, elevated blood pressure, light-headedness, fall, chest pain, toothache), minor events unrelated to the study (ankle pain, bronchitis), and cardiovascular re-hospitalisation in both groups. A single trial reported detailed adverse events which were listed in a tabular form in the supplementary material [99].

## 4 Discussion

This review shows that interventions using activity trackers may increase daily step count in older adults with chronic health conditions, with pooled estimates that in some analyses exceed the clinically meaningful change (775 steps). However, certainty in this effect estimate varied substantially across follow-up time points. In the short term and at intermediate follow‑up, the certainty of evidence was very low, meaning the true effect may be substantially different from the observed estimates. In contrast, at around 12 months, activity trackers probably increase daily step count, based on moderate certainty evidence. Evidence beyond one year remains limited and uncertain, given that it is derived from only one study. Sustainability may be influenced by declining adherence, reduced device engagement, and challenges in maintaining behaviour change.

The high level of heterogeneity observed in the short-term meta-analysis (I² = 90%) highlights important variability across included studies and should be interpreted cautiously. Several factors may explain this inconsistency. First, there was substantial variation in intervention design, with some studies using activity trackers as standalone tools, while others incorporated them within multicomponent interventions including physical activity counselling, behavioural strategies, or remote monitoring. Second, the included populations represented a wide range of chronic conditions (e.g., diabetes, COPD, stroke, and mixed conditions), which may influence responsiveness to physical activity interventions. Third, there were differences in intervention duration and timing of outcome assessment, with “short-term” endpoints varying across studies. These sources of clinical and methodological diversity likely contributed to the variability in effect estimates and reduced confidence in pooled short-term findings.

When compared directly to other active interventions, interpretation of findings of the activity trackers influence across all measured outcomes (step count, mobility, quality of life, and mental health) was limited by heterogeneity in control conditions across studies, which restricted quantitative synthesis and complicates direct comparison of effect estimates. Adverse events were generally minor and comparable between groups. The overall certainty of evidence was low to very low across most outcomes, reflecting substantial heterogeneity and methodological limitations of the included trials.

The exploratory subgroup analyses suggest that activity trackers may vary across diverse populations, although substantial heterogeneity was observed and certainty of evidence varied across subgroups. Diabetes mellitus showed the largest increase (2014 steps/day), although with very low certainty of evidence, followed by stroke (1369 steps/day), with moderate certainty of evidence and COPD (1245 steps per day) with very low certainty of evidence. Cancer and chronic conditions demonstrated moderate step increases (1153 and 1159 steps/day, respectively), with moderate and low certainty of evidence. Cardiovascular diseases and musculoskeletal conditions showed no statistically significant differences. Given that these subgroups analyses were exploratory, often based on a small number of trials, and frequently supported by low or very low certainty evidence, they should be interpreted as hypothesis‑generating rather than evidence of differential effectiveness between conditions. Additionally, our sensitivity analysis restricted to high-quality studies yielded a conservative overall estimate of 1498 steps/day, suggesting that methodological rigor attenuates but does not eliminate the observed effect.

Our findings are broadly consistent with previous evidence showing that technology-based interventions can increase physical activity among older adults, although much of the existing evidence has focused on short‑term effects and has been limited by low certainty.

Prior reviews [1,[36], [37], [38]] have reported small to moderate effects of wearable activity trackers on physical activity, often over intervention periods of up to 12 weeks, with benefits more apparent when combined with behavioural strategies.

Compared with this literature, our review extends current knowledge by focusing exclusively on RCTs in older adults with chronic health conditions and by examining outcomes across longer follow‑up periods. While short‑term effects on step count were characterised by very low certainty and substantial heterogeneity, activity trackers probably increase daily step count at around 12 months, based on moderate certainty evidence. In contrast to reviews comparing trackers primarily with minimal controls, we found no clear differences when activity trackers were compared with other active interventions, suggesting that they may perform similarly to conventional physical activity strategies rather than providing additional benefit.

### 4.1 Strengths and limitations

This review followed PRISMA guidelines with pre-registered protocol (PROSPERO) and comprehensively searched six major databases without limitations on language or publication date. We evaluated varied settings, a wide range of devices and outcomes at several time points, including follow-up to two years after the intervention, and most included studies had high methodological quality (mean PEDro score ≥ 6). Despite these advantages, our limitations included missing studies from low- and middle-income nations. Furthermore, we were unable to conduct subgroup analyses according to the kind of intervention (wearables vs smartphone apps (single study reported the use of smartphone app), walking tests vs functional outcome measures or sensitivity analyses for risk of bias because of the insufficient number of studies, particularly for secondary outcome comparison with another active intervention. Publication bias was not explored for all outcomes because of the fewer number of included studies (<10) [104]. Long term sustainability was not evaluated for all outcomes, health conditions and comparisons because of limited number of trials posing challenges to the applicability of the interventions. Although this review aimed to evaluate both activity trackers and smartphone applications, the available evidence was primarily derived from wearable devices such as pedometers, accelerometers, and commercial fitness trackers (e,g., Fitbit etc.). Only three studies [[66], [78], [98]] evaluated a smartphone application paired with an activity tracker. Therefore, the conclusions of this review primarily reflect the effectiveness of activity trackers, and evidence for smartphone applications remains very limited. As such, any inference regarding smartphone-based interventions should be interpreted with caution, and further research is needed to determine their effectiveness in older adults with health conditions.These limitations highlight the need for further research on different health conditions with long term follow-ups.

### 4.2 Implications for policymakers and clinicians

Majority (37 out of the 44) included studies were conducted in high-income countries, representing a critical evidence gap that limits generalisability of findings. The WHO identifies real-world obstacles to widespread adoption of technology-based gadgets in lower- and middle-income settings [105]. Accordingly, our findings are most applicable to high-income contexts where activity trackers are feasible and cost-effective adjuncts to physical activity promotion. These results should not be extrapolated to resource-constrained settings without context-specific implementation research, which accounts for most of the world’s population. Future trials are urgently needed in low- and middle-income countries to evaluate whether activity trackers remain effective and cost-effective when implemented under real-world conditions with limited technical support, variable internet connectivity, and competing health priorities. Additionally, high-quality, long-term studies are needed in understudied populations, particularly older adults with chronic conditions in diverse socioeconomic contexts. The finding that most high-quality trials integrated activity trackers within broader behavioural or psychological interventions suggests that standalone device distribution is unlikely to be effective; future research should clarify whether such multicomponent approaches are feasible and sustainable in resource-limited settings.

Activity trackers may be considered as an option to support physical activity in clinical and public health settings, given the moderate evidence of sustained step count increases at one-year follow-up. However, implementation should be selective and context dependent. Short-term increase in step count with very low certainty evidence should be interpreted cautiously and may not appear to translate reliably into improvements in mobility, quality of life, or mental health. This pattern suggests that activity trackers primarily function as tools to support physical activity monitoring and behaviour change rather than as comprehensive health interventions. Given the substantial heterogeneity across studies, programs implementing activity trackers should include robust monitoring of adherence and real-world effectiveness, particularly beyond 12 months where evidence is sparse. Cost-effectiveness analyses and long-term follow-up studies are essential before widespread policy adoption. Clinicians should counsel patients that activity tracker benefits are limited to increasing step count, and expectations for secondary health gains should be managed accordingly.

## 5 Conclusion

Interventions using activity trackers increased physical activity in the short-long term with very low to moderate certainty evidence when compared to no or minimal interventions. Evidence remains uncertain at intermediate and very long-term follow-ups. No benefits were reported for mobility, quality of life and mental health outcomes with low to high certainty evidence. Subgroup analyses suggest activity trackers may improve physical activity across metabolic, neurological, and pulmonary conditions based on moderate to very low certainty evidence.

## Ethics approval

The study did not require Research Ethics Committee approval as it is a systematic review.

## Declaration of generative AI and AI-assisted technologies in the writing process

The authors declare that generative AI tools were used sparingly and solely for language refinement and improving sentence clarity. AI tools were not used to generate scientific content, interpret data, draw conclusions, or influence the study design. All content was critically reviewed, edited, and approved by the authors, who take full responsibility for the accuracy, originality, and integrity of the manuscript.

## Source(s) of support

This research received no funding. Nusrat Hamdani is supported by Research Training Program (RTP) Fees offset scholarship funded by the Australian Government.

## Data availability

Available on request.

## CRediT authorship contribution statement

**Nusrat Hamdani:** Writing – review & editing, Writing – original draft, Visualization, Validation, Software, Resources, Project administration, Methodology, Investigation, Funding acquisition, Formal analysis, Data curation, Conceptualization. **Bruno T Saragiotto:** Conceptualization, Methodology, Validation, Formal analysis, Supervision, Writing – review & editing. **Peter Stubbs:** Methodology, Investigation, Data curation, Formal analysis, Writing – review & editing. **Juliana Oliveira:** Conceptualization, Methodology, Supervision, Writing – review & editing. **Anne Tiedemann:** Writing – review & editing. **Catherine Sherrington:** Writing – review & editing. **Paloma Santana:** Investigation, Data curation. **Gabriel Ferretti:** Investigation, Data curation. **Arianne Verhagen:** Methodology, Supervision, Writing – review & editing. **Poonam Mehta:** Methodology, Formal analysis, Supervision, Writing – review & editing.

## Declaration of competing interest

On behalf of all authors, the corresponding author states that there is no conflict of interest.

## Appendix Supplementary materials

Image, application 1

Image, application 2

Image, application 3

Image, application 4

Image, application 5

Image, application 6
