# Supplementary material for: Effectiveness of interventions using activity trackers and smartphone applications for increasing physical activity in older adults with health conditions: a systematic review with meta-analysis
Source: J Frailty Aging. 2026 Aug 31;15(4):100180. doi: 10.1016/j.tjfa.2026.100180 (PMC13558122; doi:10.1016/j.tjfa.2026.100180)
Supplement: Supplementary file 1 [file mmc1.docx]

(*search strategy*)

**Embase (2506)**

1 pedometer*.mp.

2 ((step* or walk*) adj3 (count* or meter* or daily)).mp.

3 ((activ* or exercise) adj3 (track* or monitor* or device*)).mp.

4 (Fitbit or garmin or step watch).mp.

5 (manpo meter or manpo-meter or manpometer or step meter or stepmeter or step meters).mp.

6 activity tracker/

7 fitness tracker/

8 mobile application/

9 ((smart phone or smartphone or mobile or mobile fitness) adj4 app*).mp. [mp=title, abstract, heading word, drug trade name, original title, device manufacturer, drug manufacturer, device trade name, keyword heading word, floating subheading word, candidate term word]

10 1 or 2 or 3 or 4 or 5 or 6 or 7 or 8 or 9

11 (elderly or senior*or geriatric*).mp.

12 (older adj (adult* or people or person*1)).mp.

13 *Aged/ or *"Aged, 80 and over"/

14 11 or 12 or 13

15 (Randomized Controlled Trial or Controlled Clinical Trial or Pragmatic Clinical Trial or Clinical Study or Adaptive Clinical Trial or Equivalence Trial).mp.

16 Random Allocation/

17 Control Groups/

18 Double-Blind Method/

19 Single-Blind Method/

20 Placebos/

21 randomized controlled trial/

22 controlled clinical trial/

23 (clinical* adj5 trial*).mp.

24 (randomi?ed adj5 trial).mp.

25 15 or 16 or 17 or 18 or 19 or 20 or 21 or 22 or 23 or 24

26 10 and 14 and 25

**Medline (2,925)**

1	pedometer*.mp.

2	((step* or walk*) adj3 (count* or meter* or daily)).mp.

3	((activ* or exercise) adj3 (track* or monitor* or device*)).mp.

4	(Fitbit or garmin or step watch).mp.

5	(manpo meter or manpo-meter or manpometer or step meter or stepmeter or step meters).mp.

6	activity tracker/

7	fitness tracker/

8	mobile application/

9	((smart phone or smartphone or mobile or mobile fitness) adj4 app*).mp.

10	1 or 2 or 3 or 4 or 5 or 6 or 7 or 8 or 9

11	(elderly or senior*or geriatric*).mp.

12	(older adj (adult* or people or person*1)).mp.

13	exp Aged/ not Adolescent.mp.

14	*Aged/ or *"Aged, 80 and over"/

15	11 or 12 or 13 or 14

16	(Randomized Controlled Trial or Controlled Clinical Trial or Pragmatic Clinical Trial or Clinical Study or Adaptive Clinical Trial or Equivalence Trial).pt.

17	Random Allocation/

18	Control Groups/

19	Double-Blind Method/

20	Single-Blind Method/

21	Placebos/

22	randomized controlled trial/

23	controlled clinical trial/

24	(random* or RCT or RCTs).mp.

25	(controlled adj5 (trial* or stud*)).mp.

26	(clinical* adj5 trial*).mp.

27	(randomi?ed adj5 trial).mp.

28	16 or 17 or 18 or 19 or 20 or 21 or 22 or 23 or 24 or 25 or 26 or 27

29	10 and 15 and 28

**CINAHL (411)**

| S29 | S10 AND S15 AND S28 |  |
| --- | --- | --- |
| S28 | S16 OR S17 OR S18 OR S19 OR S20 OR S21 OR S22 OR S23 OR S24 OR S25 OR S26 OR S27 |  |
| S27 | (MH "Randomized Controlled Trials") OR (MH "Clinical Trials") OR "(randomi#ed N5 trial)" |  |
| S26 | (MH "Clinical Trials") OR (MH "Randomized Controlled Trials") OR "(clinical* N5 trial*)" |  |
| S25 | (MH "Randomized Controlled Trials") OR (MH "Clinical Trials") OR "(controlled N5 (trial* or stud*))" |  |
| S24 | (MH "Randomized Controlled Trials") OR "(random* or RCT or RCTs)" |  |
| S23 | (MH "Randomized Controlled Trials") OR (MH "Clinical Trials") OR "controlled clinical trial[pt]" |  |
| S22 | (MH "Randomized Controlled Trials") OR (MH "Clinical Trials") OR "randomized controlled trial or rct" |  |
| S21 | (MH "Placebos") OR "placebos [mesh]" |  |
| S20 | (MH "Single-Blind Studies") OR "single-blind method" |  |
| S19 | (MH "Double-Blind Studies") OR "double-blind method" |  |
| S18 | (MH "Control Group") OR "control groups, randomized" |  |
| S17 | "random allocation" |  |
| S16 | (MH "Randomized Controlled Trials") OR (MH "Clinical Trials") OR (MH "Clinical Trial Registry") OR "(Randomized Controlled Trial or Controlled Clinical Trial or Pragmatic Clinical Trial or Clinical Study or Adaptive Clinical Trial or Equivalence Trial).pt." |  |
| S15 | S11 OR S12 OR S13 OR S14 |  |
| S14 | (MH "Aged, 80 and Over") OR "*Aged/ or *"Aged, 80 and over"" |  |
| S13 | "exp Aged/ not Adolescent" |  |
| S12 | "(older N (adult* or people or person*1))" |  |
| S11 | (MH "Geriatrics") OR (MH "Frail Elderly") OR "(elderly or senior*or geriatric*)" |  |
| S10 | S1 OR S2 OR S3 OR S4 OR S5 OR S6 OR S7 OR S8 OR S9 |  |
| S9 | (MH "Mobile Applications") OR "((smart phone or smartphone or mobile or mobile fitness) N4 app*)" |  |
| S8 | (MH "Mobile Applications") OR "mobile applications" |  |
| S7 | (MH "Fitness Trackers") OR (MH "Wearable Sensors") OR "activity tracker or fitness trackers or wearable technology" |  |
| S6 | "(manpo meter or manpo-meter or manpometer or step meter or stepmeter or step meters)" |  |
| S5 | "(Fitbit or garmin or step watch)" |  |
| S4 | "((active* or exercise) N3 (track* or monitor* or device*))" |  |
| S3 | "((step* or walk*) N3 (count* or meter* or daily))" OR (MH "Step") OR (MH "Walking Speed") |  |
| S2 | "((step* or walk*) N3 (count* or meter* or daily))" |  |
| S1 | (MH "Pedometers") OR "pedometer" |  |

**Psycinfo (123)**

| S38 AND S42 AND S53 | | |
| --- | --- | --- |
| S43 OR S44 OR S45 OR S46 OR S47 OR S48 OR S49 OR S50 OR S51 OR S52 | | |
| controlled N5 (trial* or stud*) | | |
| (random* or RCT or RCTs) | | |
| DE "Placebo" | | |
| Single-Blind Method | | |
| Double-Blind Method | | |
| DE "Clinical Trials" | | |
| DE "Experiment Controls" | | |
| DE "Randomized Controlled Trials" OR DE "Randomized Clinical Trials" | | |
| (Randomized Controlled Trial or Controlled Clinical Trial or Pragmatic Clinical Trial or  Clinical Study or Adaptive Clinical  Trial or Equivalence Trial) | | |
| (Randomized Controlled Trial or Controlled Clinical Trial or Pragmatic Clinical Trial or  Clinical Study or Adaptive Clinical  Trial or Equivalence Trial) | | |
| S39 OR S40 OR S41 | | |
| DE "Older Adulthood" NOT ( DE "Adolescent Development" OR DE "Adolescent Behavior" OR DE "  Adolescent Characteristics" OR DE "Adolescent Health" OR DE "Early Adolescence" OR  DE "Late Adolescence" ) | | |
| older N (adult* or people or person*) | | |
| elderly or senior*or geriatric* | | |
| S27 OR S28 OR S29 OR S30 OR S31 OR S32 OR S33 OR S34 OR S35 OR S36 OR S37 | | |
| ("smart phone" or smartphone or "mobile phone" or "cell phone") N4 app* | | |
| DE "Mobile Applications" OR DE "Smartphones" OR DE "Mobile Phones" OR DE "Mobile Devices" | | |
| fitness tracker | | |
| activity tracker | | |
| "manpo meter" or "manpo-meter" or manpometer or "step meter*" or stepmeter* | | |
| Fitbit or garmin or "step watch" | | |
| ((activ* or exercise) N3 (track* or monitor* or device*) | | |
| (step* or walk*) N3 (count* or meter* or daily) | | |
| pedometer* | | |
| MA pedometer | | |
| TX pedometer | | |
|  | | |
| **Sportdiscus (68)**  S27 | S11 AND S16 AND S26 |  |
| S26 | S17 OR S18 OR S19 OR S20 OR S21 OR S22 OR S23 OR S24 OR S25 |  |
| S25 | controlled N5 (trial* or stud*) |  |
| S24 | (random* or RCT or RCTs) |  |
| S23 | DE "Placebo" |  |
| S22 | Single-Blind Method |  |
| S21 | Double-Blind Method |  |
| S20 | DE "Clinical Trials" |  |
| S19 | DE "Experiment Controls" |  |
| S18 | DE "Randomized Controlled Trials" OR DE "Randomized Clinical Trials" |  |
| S17 | (Randomized Controlled Trial or Controlled Clinical Trial or Pragmatic Clinical Trial or Clinical Study or Adaptive Clinical Trial or Equivalence Trial) |  |
| S16 | S12 OR S13 OR S14 OR S15 |  |
| S15 | DE "Older Adulthood" NOT ( DE "Adolescent Development" OR DE "Adolescent Behavior" OR DE "Adolescent Characteristics" OR DE "Adolescent Health" OR DE "Early Adolescence" OR DE "Late Adolescence" ) |  |
| S14 | DE "Older Adulthood" NOT ( DE "Adolescent Development" OR DE "Adolescent Behavior" OR DE "Adolescent Characteristics" OR DE "Adolescent Health" OR DE "Early Adolescence" OR DE "Late Adolescence" ) |  |
| S13 | older N (adult* or people or person*) |  |
| S12 | elderly or senior*or geriatric* |  |
| S11 | S1 OR S2 OR S3 OR S4 OR S5 OR S6 OR S7 OR S8 OR S9 OR S10 |  |
| S10 | ("smart phone" or smartphone or "mobile phone" or "cell phone") N4 app* |  |
| S9 | DE "Mobile Applications" OR DE "Smartphones" OR DE "Mobile Phones" OR DE "Mobile Devices" |  |
| S8 | fitness tracker |  |
| S7 | activity tracker |  |
| S6 | "manpo meter" or "manpo-meter" or manpometer or "step meter*" or stepmeter* |  |
| S5 | "manpo meter" or "manpo-meter" or manpometer or "step meter*" or stepmeter* |  |
| S4 | Fitbit or garmin or "step watch" |  |
| S3 | ((activ* or exercise) N3 (track* or monitor* or device*) |  |
| S2 | (step* or walk*) N3 (count* or meter* or daily) |  |
| S1 | pedometer* |  |

**PEDRo (329)**

1. pedometer (AB TI) AND gerontology (Subdiscipline) AND clinical trial [method] Results:67
2. step count (AB TI) AND gerontology (Subdiscipline) AND clinical trial [method] 88
3. step meter (AB TI) AND gerontology (Subdiscipline) AND clinical trial [method]91
4. step daily (AB TI) AND gerontology (Subdiscipline) AND clinical trial [method] 131
5. walk count (AB TI) AND gerontology (Subdiscipline) AND clinical trial [method] 9
6. walk meter (AB TI) AND gerontology (Subdiscipline) AND clinical trial [method] 46
7. activity track (AB TI) AND gerontology (Subdiscipline) AND clinical trial [method]
8. activity monitor (AB TI) AND gerontology (Subdiscipline) AND clinical trial [method]
9. activity device* (AB TI) AND gerontology (Subdiscipline) AND clinical trial [method]38
10. Fitness tracker* (AB TI) AND gerontology (Subdiscipline) AND clinical trial [method]4
11. fitbit (AB TI) AND gerontology (Subdiscipline) AND clinical trial [method]10
12. garmin (AB TI) AND gerontology (Subdiscipline) AND clinical trial [method]0
13. Smartphone applications AND gerontology (Subdiscipline) AND clinical trial [method]1
14. Mobile application* AND gerontology (Subdiscipline) AND clinical trial [method]7
15. Mobile health AND gerontology (Subdiscipline) AND clinical trial [method]12
16. Wearable*AND gerontology (Subdiscipline) AND clinical trial [method]15
