# Supplementary material for: Effectiveness of interventions using activity trackers and smartphone applications for increasing physical activity in older adults with health conditions: a systematic review with meta-analysis
Source: J Frailty Aging. 2026 Aug 31;15(4):100180. doi: 10.1016/j.tjfa.2026.100180 (PMC13558122; doi:10.1016/j.tjfa.2026.100180)
Supplement: Supplementary file 3 [file mmc3.docx]

We thank the Editor-in-Chief, and external reviewers for their constructive comments. For ease of review, all comments and corresponding author responses are presented in the table below. All revisions in the manuscript have been highlighted in Yellow. We appreciate the careful review and believe these revisions have substantially strengthened the manuscript.

| **Reviewer 1 comments:** | **Authors’ responses** |
| --- | --- |
| No1., the short‑term meta-analysis shows extremely high heterogeneity (I² = 90%), yet the discussion provides limited explanation. Additional interpretation regarding potential sources such as variation in intervention components, health conditions, or follow‑up timing would improve transparency (heterogeneity explanation). | We thank the reviewer for this important observation. We agree that the high heterogeneity (I² = 90%) in the short-term meta-analysis warrants further clarification. We have revised the Discussion to better explain potential sources of heterogeneity. It now reads as highlighted second para:  “The high level of heterogeneity observed in the short-term meta-analysis (I² = 90%) highlights important variability across included studies and should be interpreted cautiously. Several factors may explain this inconsistency. First, there was substantial variation in intervention design, with some studies using activity trackers as standalone tools, while others incorporated them within multicomponent interventions including physical activity counselling, behavioural strategies, or remote monitoring. Second, the included populations represented a wide range of chronic conditions (e.g., diabetes, COPD, stroke, and mixed conditions), which may influence responsiveness to physical activity interventions. Third, there were differences in intervention duration and timing of outcome assessment, with “short-term” endpoints varying across studies. These sources of clinical and methodological diversity likely contributed to the variability in effect estimates and reduced confidence in pooled short-term findings.” |
| No 2., although the review aims to evaluate both activity trackers and smartphone applications, only one included study used a smartphone app. The conclusions should therefore avoid overgeneralization and clearly distinguish the strength of evidence for each technology. | We appreciate the reviewer’s comment and agree that the conclusions should more clearly reflect the evidence base. Although our review aimed to assess both activity trackers and smartphone applications, only one included study evaluated a smartphone app. We have revised the manuscript to avoid overgeneralisation and now explicitly state that the findings are primarily driven by evidence on wearable activity trackers. We also clarify that conclusions regarding smartphone applications are limited and should be interpreted with caution due to insufficient evidence.  Revised and added a highlighted section under “Strengths and limitations” section under “Discussion” which reads as:  “Although this review aimed to evaluate both activity trackers and smartphone applications, the available evidence was primarily derived from wearable devices such as pedometers, accelerometers, and commercial fitness trackers (e,g., Fitbit etc). Only one included study^1^ evaluated a smartphone application paired with an activity tracker (Fitbit) . Therefore, the conclusions of this review primarily reflect the effectiveness of activity trackers, and evidence for smartphone applications remains very limited. As such, any inference regarding smartphone-based interventions should be interpreted with caution, and further research is needed to determine their effectiveness in older adults with health conditions.” |
| No 3., long‑term effects beyond 12 months remain uncertain, but the discussion does not sufficiently address factors that may influence sustainability, such as adherence, device usage decline, or behavioural maintenance in older adults. Adding brief consideration of these issues would enhance the applicability of the findings (long‑term follow‑up). | We thank the reviewer for highlighting this important point. We agree that factors influencing very long-term sustainability beyond one year were not sufficiently discussed. The Discussion has been updated to acknowledge that evidence on very long-term effectiveness is limited, with only a single study contributing data at extended follow-up. The highlighted section in Discussion para.1 line no.8-10 now reads as:  Evidence beyond one year remains limited and uncertain, given that it is derived from only one study. Sustainability may be influenced by declining adherence, reduced device engagement, and challenges in maintaining behaviour change. |

References:

1.	Duscha BD, Piner LW, Patel MP, et al. Effects of a 12-Week mHealth Program on FunctionalCapacity and Physical Activity in Patients With PeripheralArtery Disease. Randomized Controlled Trial

Research Support, Non-U.S. Gov't. *Am J Cardiol*. 09 01 2018;122(5):879-884. doi:<https://dx.doi.org/10.1016/j.amjcard.2018.05.018>
