# Supplementary material for: Effectiveness of interventions using activity trackers and smartphone applications for increasing physical activity in older adults with health conditions: a systematic review with meta-analysis
Source: J Frailty Aging. 2026 Aug 31;15(4):100180. doi: 10.1016/j.tjfa.2026.100180 (PMC13558122; doi:10.1016/j.tjfa.2026.100180)
Supplement: Supplementary file 4 [file mmc4.docx]

Description of included studies

| S.no | Study | Participants | Intervention Group | Control Group | Outcomes |  |
| --- | --- | --- | --- | --- | --- | --- |
| 1. | *Alonso-Dominguez et al.* 2019  (Spain) | **N:** 204  **Mean age (SD) control group:** 60.4 (8.4)  **Mean age (SD) intervention group:** 60.8 (7.8)  **Inclusion criteria:**  *-Age:* 25-70 years of age  *-Health Condition:* T2DM  **-***Source of recruitment:*  primary health care center  -*Mobility status:* Able to ambulate independently  -*Physical activity status:* Not mentioned | **Intervention:**  Nurse guide 4 km walks, smartphone app, PA & diet counselling  **Type of activity tracker:**  pedometer (Omron HJ-321 Tri axis)  **Goal:** walk 10,000 steps daily  **Intervention duration (weeks)***:*13 | PA and diet counselling | **Physical activity:** Steps/day, measured with a pedometer worn on the waist |  |
| 2. | *Arbillaga-Etxarri et al.* 2018  (Spain) | **N:** 407  **Mean age (SD) control group:** 69±8  **Mean age (SD) intervention group:** 68±9  **Inclusion criteria:**  *-Age:* ⩾ 45 years of age  *-Health Condition:* COPD  **-***Source of recruitment:*  Primary and hospital setting  -*Mobility status:* Able to ambulate independently  -*Physical activity status:* Not currently enrolled in a structured exercise program | **Intervention:**  Urban Training consisted of a baseline motivational interview, advice to walk on urban trails designed for COPD patients in outdoor public spaces and other optional components for feedback, motivation, information and support (pedometer, calendar, physical activity brochure, website, phone text messages, walking groups and a phone number).  **Type of activity tracker:**  Pedometer type not mentioned  **Goal:** Patient tailored goals  **Intervention duration (weeks)***:*52 | General health counselling and the European Lung Foundation (ELF) information brochure “Living an active life with COPD”, which recommends ⩾30 min moderate physical activity⩾5 days per week. | **Physical activity:** Steps/day, measured with a pedometer  **Mobility:** 6-minute walk test (6MWT)  **Quality of life:** Health-related quality of life CAT (COPD assessment test)  **Mental health:** HAD (anxiety and depression score) – Depresource of recruitment, but ssion |  |
| 3. | *Ashizawa et al.* 2022  (Japan) | **N:** 61  **Mean age (SD) control group:** 70.3(7.7)  **Mean age (SD) intervention group:** 72.3(8.9)  **Inclusion criteria:**  *-Age:* ⩾ 50 years of age  *-Health Condition:* Minor Ischemic Stroke  **-***Source of recruitment:*  Hospital  -*Mobility status:* not mentioned  -*Physical activity status:* not mentioned | **Intervention:**  Education on reducing sedentary behaviour (SB), goal setting for SB  after hospital discharge, and self-monitoring of SB and step count.  **Type of activity tracker:**  accelerometer (Active StyleProHJA-750C)  **Goal:** Patient tailored goals  **Intervention duration (weeks)**:26 | usual approaches to increase physical activity levels during hospitalization | **Physical activity:** Steps/day, measured with an accelerometer |  |
| 4. | *Bade et al. 2021*  *(USA)* | **N:** 40  **Mean age (SD) control group:**63.2 years (SD 9.8)  **Mean age (SD) intervention group:** 66.6 years (SD 7.3)  **Inclusion criteria:**  *-Age: ~* 65 years of age  *-Health Condition:* Stage III or IV non-small cell lung cancer (NSCLC)  **-***Source of recruitment:* Yale Thoracic Oncology Program clinic  -*Mobility status*: Able to safely walk  -*Physical activity status*: Physically inactive (<150 min/week moderate or <75 min/week vigorous activity) | **Intervention**:(1) a 15min in-person teaching session regarding the benefits of physical activity in lung cancer; (2) a Fitbit® (San Francisco, CA, United States) Flex2 accelerometer (and set-up on their phone); (3) individualized walking goals based on their average daily step count during week 1; and (4) twice daily gain-framed text messages.  **Type of activity tracker:** a FitBit® (San Francisco, CA, United States) Flex2 accelerometer (and set-up on their phone)  **Goal**: Increase physical activity safely and improve quality of life  Intervention duration (weeks)  **Intervention duration (weeks)**:12 | Usual care | **Physical activity:** Steps/day, measured with an FitBit® Flex2  **Quality of life:** European Organisation for the Research and Treatment of Cancer Quality of Life Core Questionnaire (EORTCQLQ-C30)  **Mental Health:** Patient Health Questionnaire-9 depression scale (PHQ-9) |  |
| 5. | *Brickwood et al.* 2021  (Australia) | **N:** 117  **Mean age (SD) control group:** 71.9 (6.0)  **Mean age (SD) intervention group:** 72.3 (7)  **Inclusion criteria:**  *-Age:* ⩾ 60 years of age  *-Health Condition:* Chronic Health condition  **-***Source of recruitment:* community  -*Mobility status:* Able to ambulate independently  -*Physical activity status:* not mentioned | **Intervention:**  Activity tracker + app, telephone support, daily personalised goals  **Type of activity tracker:**  accelerometer ActivPAL (TM)  **Goal:** daily personalised goals  **Intervention duration (weeks)***:* 12 | Usual care | **Physical activity:** Steps/day, measured with an accelerometer ActivPAL (TM)  **Mobility:** 6-minute walk test (6MWT)  **Quality of life:** Short Form (SF)-36 physical health summary score  **Mental Health:** Short Form (SF)-36 (mental health score) |  |
| 6. | *Bronas et al. 2024*  (USA) | **N:** 39  Mean age (SD): 61.0 (5.8)  **Inclusion criteria:**  -*Age:* 55-89 years  -*Health Condition:* high risk for cognitive decline  -*Source of recruitment: community-*dwelling, Spanish-speaking Latino adults  -*Mobility status:* NA  -*Physical activity status:* Low levels of physical activity | **Intervention:** Activity Monitor; Weekly Phone Meetings; Interactive Tools: SMS text messages; Coach Feedback  **Type of activity tracker:** Fitbit charge 4  **Goal:** to replace sedentary time with physical activity  **Intervention duration (weeks):** 6 | PA education | **Physical activity:** using a pedometer Fitbit charge 4 |  |
| 7. | *Chudowolska et al.* 2020  (Poland) | **N:** 199  **Mean age (SD) control group:** 63(7)  **Mean age (SD) intervention group:** 62 (7)  **Inclusion criteria:**  *-Age:* 50-75 years of age  *-Health Condition:* sedentary old adults with CVS risk factors  **-***Source of recruitment:* Primary care clinic  *Mobility status:* not disabled  -*Physical activity status:* Not mentioned | **Intervention:**  Nurse-led intervention, goal setting and supporting phone calls  **Type of activity tracker:** pedometer (M2Smartband, EnweiTechnology Co.Ltd., Shenzhen China)  **Goal:** minimum 7000 steps per day  **Intervention duration (weeks)***:* 13 | Daily goal of at least 7000 steps, telephone calls & support phone | **Physical activity:** Steps/day, measured with a pedometer |  |
| 8. | *Cruz et al.* 2016  (Portugal) | N: 27  Mean age (SD) control group: 64 (8)  Mean age (SD) intervention group: 69 (8) Inclusion criteria:  -Age: ≥18 years  -Health Condition: COPD  -Source of recruitment: Primary care and hospital  -Mobility status: Not reported  -Physical activity status: Not reported | **Intervention:** Usual care (pulmonary rehabilitation); physical activity behavioural intervention; pedometer; daily steps diary; education session; and telephone support  **Type of activity tracker:** Yamax Power Walker EX510  **Goal:** Weekly increase of 800 steps/day to reach recommendations of 7000 to 10,000 steps/day **Intervention duration (weeks):**12 | Usual care (pulmonary rehabilitation) | **Physical activity:** Steps/day, measured with an accelerometer (GT3X+) worn on waist  **Quality of life:** St. George's respiratory questionnaire |  |
| 9. | *Dasgupta et al.* 2017  (Canada) | **N:** 347  **Mean age (SD) control group:** 59 (11)  **Mean age (SD) intervention group:** 60.0 (11)  **Inclusion criteria:**  -*Age:* ≥18 years  -*Health Condition:* Type 2 diabetes and hypertension  -*Source of recruitment:* Primary care  -*Mobility status:* Absence of gait impairment.  -*Physical activity status:* Not reported | **Intervention:** Given a pedometer; daily steps diary; and step count prescription  **Type of activity tracker:** Yamax Digi-Walker SW701  **Goal:** Increase over baseline by 3000 steps/day over 1 year **Intervention duration (weeks):**52 | Usual care (physical activity advice) | **Physical activity:** Steps/day, measured with a pedometer (Yamax Digi-Walker SW-701) worn on waist. |  |
| 10. | *De greef et al.* 2011  (Belgium) | **N**: 92  **Mean age (SD):** 62 (9)  **Inclusion criteria:**  -*Age:* 35-75 years  -*Health Condition:* Type 2 diabetes  -*Source of recruitment:* Hospital  -*Mobility status:* No documented physical limitations  -*Physical activity status:* Not reported | **Intervention:** A 30 min face-to-face session; pedometer; daily steps diary; telephone support  **Type of activity tracker:** Yamax Digi-Walker SW200  **Goal:** Patient-tailored goals **Intervention** **duration (weeks):**24 | No intervention | **Physical activity:** Steps/day, measured with a pedometer (Yamax Digi-Walker SW-200). Pedometer placement was not reported |  |
| 11. | *De meyer et al.* 2017  (Belgium) | **N:** 92 Mean age (SD): 62 (9)  **Inclusion criteria:**  -*Age:* 35-75 years  -*Health Condition:* Type 2 diabetes  -*Source of recruitment:* Hospital  -*Mobility status:* No documented physical limitations  -*Physical activity status:* Not reported | **Intervention:** A 30 min face-to-face session; pedometer; daily steps diary; telephone support  **Type of activity tracker:** Yamax Digi-Walker SW200  **Goal:** Patient-tailored goals **Intervention duration (weeks):**24 | Education: Standard leaflet explaining the importance of physical activity in COPD | **Physical activity:** Steps/day, measured with an accelerometer (Actigraph GT3x) worn on waist  **Quality of life:** COPD assessment test (CAT) |  |
| 12. | *De Oliveira et al. 2024*  (Australia) | **N:** 35  Mean age (SD): 68.7 ±5.9  **Inclusion criteria:**  -*Age:* ≥60 years  -*Health Condition:* Type 2 diabetes and uncontrolled hypertension (high blood pressure)  -*Source of recruitment:* Outpatient Clinic of a Hospital  -*Mobility status*: NA  *-Physical activity status*: NA | **Intervention:** dietary guidance plus encouragement to increase physical activity using a pedometer  **Type of activity tracker:** HJ-321 Omron® (pedometer)  **Goal:** increase the number of their steps at each monthly visit after allocation.  **Intervention duration (weeks):**16 | Education | **Physical activity:** steps/day measured with a pedometer attached to the clothes on the waistline. |  |
| 13. | *Dohrn et al.* 2017  (Sweden) | **N:** 351  **Mean age (SD) control group:** 75.2 (4.6)  **Mean age (SD) intervention group:** 75.7 (5.8)  **Inclusion criteria:**  *-Age:* ⩾ 60 years of age  *-Health Condition:*  osteoporosis  **-***Source of recruitment:* community-dwelling  -*Mobility status:* able to walk indoors with or without support  -*Physical activity status:* not mentioned | **Intervention:**12-week balance training program with three 45-minute sessions per week  **Type of pedometer:**  Pedometer (Yamax LS2000, Yamax Corporation, Japan) and an accelerometer (ACTi graph GT1M or GT3X+, ACTi Graph).  **Goal:** minimum level of 5000 steps/day as a cut-off  **Intervention duration (weeks)***:*13 | Usual care | **Physical activity:** Steps/day, measured with a pedometer |  |
| 14. | *Dodson et al. 2025*  (USA) | **N:** 400  **Mean age (SD) control group:** 71.0 (range 65.0-89.0)  **Mean age (SD) intervention group:** 71.0 (range 65.0-91.0)  **Inclusion criteria:**  *-Age:* ≥65 years  *-Health Condition:* ischemic heart disease (hospital visit for myocardial infarction or coronary revascularization)  **-***Source of recruitment:* academic hospitals  -*Mobility status:* NA  -*Physical activity status:* no prescribed physical activity restriction | **Intervention:** mHealth-CR software on a tablet,  [1]8 monitoring,  Weekly exercise therapist telephone calls  **Type of pedometer:** Fitbit Inspire  **Goal:** Improve functional capacity and health status  **Intervention duration (weeks)**:12 | Usual care with the option to attend traditional cardiac rehabilitation | **Physical activity:** Steps/day, measured with a Fitbit Inspire  **Mobility:** 6-minute walk test (6MWT)  **Quality of life:** SF-12 physical component score [12-Item Short Form Health Survey (SF-12)]  **Mental health:** SF-12 mental component score [12-Item Short Form Health Survey (SF-12)] |  |
| 15. | *Duscha et al.* 2018  (USA) | **N:** 20  **Mean age (SD) control group:** 73.1(4.7)  **Mean age (SD) intervention group** 66.1(9.8)  **Inclusion criteria:**  *-Age:* Not reported  *-Health Condition:* Peripheral artery disease (PAD)  **-***Source of recruitment:* University  Medical Center cardiology clinics  -*Mobility status:* not reported  *Physical activity status:* Sedentary | **Intervention:**  12-week health intervention consisting of patient education, smartphones, and physical activity trackers.  **Type of activity tracker:** Fitbit Charge device (Fitbit, Inc., San Francisco, CA)  **Goal:** Patient-tailored goals  **Intervention duration (weeks)***:* 12 | Usual care | **Physical activity:** Steps/day, measured with a Fitbit Charge device (Fitbit, Inc., San Francisco, CA) |  |
| 16. | *Fanning et al.* 2022  (USA) | **N:** 30  **Mean age (SD) control group:** 68.55(8.02)  **Mean age (SD) intervention group:** 68.55(8.02)  **Inclusion criteria:**  *-Age:* 55–85 years of age  *-Health Condition:*  Older adults with chronic pain  **-***Source of recruitment:*  Community  -*Mobility status:* Not reported  -*Physical activity status:* did not engage in regular resistance training and/or more than 20 min of aerobic exercise on 2 or more days per week in the previous 6 months | **Intervention:**  mHealth mediated active intervention, which focused on reducing caloric intake and increasing steps across the day  **Type of activity tracker:**  Fitbit Alta activity monitor and Fitbit app  **Goal:** to break sustained siting time  **Intervention duration (weeks)***:*12 | waitlist control condition. | **Physical activity:** Steps/day, measured with a Fitbit |  |
| 17. | *Gell et al. 2022*  *(USA)* | **N:** 39  Mean age (SD): 70.4 (5.7)  **Inclusion criteria:**  -*Age*: ⩾ 60 years  -*Health Condition*: cancer survivors  -*Source of recruitment:* rural community  -*Mobility status*: minimal participation in  exercise  -*Physical activity status:* <150 min/week MVPA | **Intervention:** Enhance Fitness (tele-EF) exercise program; included aerobic, strength, and balance training; led by certified instructors and conducted for 1 hour, 3 days a week  **Type of activity tracker:** activPAL4 accelerometer  **Goal:** Increase PA  **Intervention duration (weeks):**16 | Waitlist control | **Physical activity:** steps/day measured with an accelerometer  **Mobility:**5 times sit to stand |  |
| 18. | *Grau-Pellicer et al.* 2020  (Spain) | **N:** 41  **Mean age (SD) control group:** 68.53(11.53)  **Mean age (SD) intervention group:** 62.96 (11.87)  **Inclusion criteria:**  *-Age:* ⩾ 18 years of age  *-Health Condition:* Stroke  **-***Source of recruitment:* Hospital  -*Mobility status:* functional ambulation classification (FAC) ≥3; Barthel Index ≥45  -*Physical activity status:* not reported | **Intervention:** Multimodal Rehabilitation Program (MMRP) using mHealth app for Aerobic exercises  Task-oriented exercises  Balance exercises  Stretching exercises  Home-based ambulation program  **Type of activity tracker:**  Pedometer + mhealth app.  **Goal:** To reach PA levels as recommended by the World Health Organization (WHO) of 150 m/wk of moderate PA  **Intervention duration (weeks)***:*12 | Another Physical activity intervention in the form of exercises and a conventional rehabilitation program | **Physical activity:** Steps/day, measured with a pedometer  **Mobility:**6-minute walk test (6MWT)  **Quality of life:** EuroQol 5-Dimension 5-Level (EQ5D-5 L questionnaire) |  |
| 19. | *Hassett et al.* 2020  (Australia) | **N:** 300  **Mean age (SD) control group:** 73(15)  **Mean age (SD) intervention group:** 70(18)  **Inclusion criteria:**  *-Age:* ⩾ 60years  *-Health Condition:* Patients admitted for neurological rehabilitation  **-***Source of recruitment:* Hospital  -*Mobility status:* reduced mobility-*Physical activity status:* Not reported | **Intervention:** Digitally enabled rehabilitation prescribed by physiotherapists.  **Type of activity tracker:** Fitbit  **Goal:** patient tailored goals  **Intervention duration (weeks)***:* 13 | Usual care | **Physical activity:** Steps/day, measured by *Fitbit*  **Mobility:** de morton mobility index **Mental Health**: Short Form 6 dimensions questionnaire (mental health domain) |  |
| 20. | *Heron et al.* 2017  (United Kingdom) | **N:** 28  **Mean age (SD) control group:** 76.2  **Mean age (SD) intervention group:**63  **Inclusion criteria:**  *-Age:>18* years of age  *-Health Condition:* TIA & minor stroke  **-***Source of recruitment:* Hospital  -*Mobility status: not reported*  -*Physical activity status: not reported* | **Intervention:** ‘The Healthy Brain Rehabilitation Manual’ + pedometer  **Type of pedometer:**  Pedometer  **Goal:** Patient-tailored goals  **Intervention duration (weeks)***:*4 | Usual care | **Physical activity:** Steps/day, measured with a pedometer  **Mobility:** 2-minute walk test (2MWT)  **Quality of life**: EuroQol 5-Dimension 5-Level (EQ5D-5 L questionnaire)  **Mental health:** Hospital Anxiety and Depression (HADs) questionnaire |  |
| 21. | *Hill et al. 2024*  *(Australia)* | **N:** 22  Mean age (SD) Control group: 71.7 ± 7.5  Mean age (SD) Intervention group 69.6 ± 9.2  **Inclusion criteria:**  -*Age*: ≥50 years  -*Health Condition*: chronic disease  -*Source of recruitment*: a rehabilitation clinic at a hospital and a general practitioner (GP) clinic  -*Mobility status*: able to independently ambulate with or without an aid  -*Physical activity status:* no prescribed physical activity restriction | **Intervention:** Evidence-based strength and balance exercise class.  Weekly wellness activity  Fitbit™ (Fitbit™ Inspire 2) or pedometer for step count monitoring  **Type of activity tracker:** Fitbit™ (Fitbit™ Inspire 2) or pedometer  **Goal:** Increase physical activity and social engagement  **Intervention duration (weeks):**12 | Healthy Aging for Midlife and Beyond workbook and Fitbit™ or pedometer | **Physical activity:** steps/day measured with a Fitbit™ or pedometer  **Mobility:** Timed Up and Go test (TUG)  **Quality of life:** Health-Related Quality of Life (HRQOL) by EQ-5D-5L and Visual Analog Scale EQ-VAS |  |
| 22. | *Hinrichs et al.* 2016  (Switzerland) | **N:** 209  **Mean age (SD) control group:** 78(75.7)  **Mean age (SD) intervention group:** 76(71.7)  **Inclusion criteria:**  *-Age:* ⩾50 years  *-Health Condition:* Chronic disease  **-***Source of recruitment:* Community  -*Mobility status:* mobility limitation  *Physical activity status:* Did not participate in regular PA (exercises, sports, or leisure activities that cause sweating or harder breathing for ≥2h/wk or walking outdoors for ≥4h/wk | **Intervention:** Given a pedometer; daily steps dairy  **Type of activity tracker:** pedometer (Walking style Pro, Omron Healthcare, Kyoto, Japan)  **Goal:** Weekly increase of daily steps by 10% to reach an average of 10,000 steps/day  **Intervention duration (weeks)***:* 12 | Usual care; Education | **Physical activity:** Steps/day, measured with a pedometer  **Mobility:** Timed Up and Go test (TUG)  **Quality of life:** Health-related quality of life (Medical Outcomes Study 8-item Short-Form Survey)-Physical Component Summary  **Mental Health:** Health-related quality of life (Medical Outcomes Study 8-item Short-Form Survey) - Mental Component Summary |  |
| 23. | *Hornikx et al.* 2015  (Belgium) | **N:** 27  **Mean age (SD) control group:** 68 (6)  **Mean age (SD) intervention group:** 66 (7)  **Inclusion criteria:**  -*Age:* ≥40 years  -*Health Condition:* COPD  -*Source of recruitment:* Hospital  -*Mobility status:* Not reported  -*Physical activity status:* Not reported | **Intervention:** Given a pedometer; three telephone physical activity counselling sessions per week  **Type of activity tracker:** Fitbit Ultra  **Goal:** Individualised goals with the aim of maximising physical activity levels  **Intervention duration (weeks):**4 | Physical activity advice | **Physical activity:** Steps/day, measured with an accelerometer (Dynaport Move Monitor) worn on waist  **Quality of life:** COPD assessment test (CAT) |  |
| 24. | *Kanai et al.* 2018  (Japan) | **N:** 55  **Mean age (SD) control group:** 62.9 (9.1)  **Mean age (SD) intervention group:** 66.8 (10.0)  **Inclusion criteria:**  *-Age: < 80 yrs*  *-Health Condition:* Acute ischemic stroke  **-***Source of recruitment:*  *Hospital*  -*Mobility status:* able to walk in 10m walk test  -*Physical activity status:* not reported | **Intervention:** AT (activity tracker) based feedback + goal setting  **Type of activity tracker:** Fitbit One (Fitbit, Inc., San Francisco, CA, USA)  **Goal:** personalised goal setting  **Intervention duration (weeks)***:* 12 days | Usual care | **Physical activity:** Steps/day,  measured with an accelerometer |  |
| 25. | *Kirk et al.* 2009  (United Kingdom) | **N:** 73  **Mean age (SD) control group:** 59 (10)  **Mean age (SD) intervention group:** 63 (11)  **Inclusion criteria:**  -*Age:* Not reported  -*Health Condition:* Type 2 diabetes  -*Source of recruitment:* Primary care  -*Mobility status:* Not reported  -*Physical activity status:* Not reported | **Intervention:** A written self-instructional workbook based on the transtheoretical model; a pedometer; a 12- week walking plan and guide to local physical activity opportunities; telephone calls.  **Type of activity tracker:** Not specified  **Goal:** To increase physical activity to meet ACSM health recommendations  **Intervention duration (weeks):** 24 | Exercise and diabetes leaflet | **Physical activity:** Steps/day |  |
| 26. | *Kitamura et al. 2024*  *(Japan)* | **N:** 52  Mean age (SD): 79.8±8.8  **Inclusion criteria:**  -*Age*: ≥65 years  -*Health Condition*: need long-term care services (chronic conditions)  -*Source of recruitment*: day care center  -*Mobility status*: able to walk, either with or without aids  *-Physical activity status*: NA | **Intervention:** education on physical activity  and self-monitoring such as goal setting, self-management and feedback  **Type of activity tracker:** a tri-axial accelerometer (Active Style Pro  HJA-750C, OMRON, Kyoto, Japan)  **Goal:** Patient-tailored goals  **Intervention duration (weeks):**5 | Pamphlets and accelerometer to measure steps but no feedback or education for goals | **Physical activity:** steps/day measured with an accelerometer attached to the waist  **Quality of life:** EuroQol 5-Dimension 5-Level (EQ 5D 5L) |  |
| 27. | *Kenfield et al.* 2019  (Australia) | **N:** 76  **control group:** Median 65 IQR (60,69)  **intervention group:** Median 66 IQR (61,68)  **Inclusion criteria:**  -*Age*: 51-79 years  -*Health Condition:* Prostate cancer  -*Source of recruitment:* Hospital  -*Mobility status:* Ability to walk unassisted  -*Physical activity status:* Not reported | **Intervention:** Website, Fitbit One, and text messaging to facilitate adoption of eight behaviours: vigorous activity, smoking cessation, and six diet improvements.  **Type of activity tracker:** Fitbit  **Goal:** Personalised goal setting of 3+hr/week of vigorous PA  **Intervention duration (weeks):** 12 | Usual care | **Physical activity:** Steps/day, measured with a Fitbit |  |
| 28. | *Li et al.* 2020  (China) | **N:** 51  **Mean age (SD) control group:** 64.8 (9)  **Mean age (SD) intervention group**: 65.0 (8)  **Inclusion criteria:**  -*Age:* ≥50 years  -*Health Condition:* Knee osteoarthritis (OA)  -*Source of recruitment:* medical centre, hospitals and community-based services  -*Mobility status:* Not specified  -*Physical activity status:* Not specified | **Intervention:** Group education session  Use of a Fitbit Flex-2 wristband  Access to FitViz (a Fitbit-compatible app)  4 biweekly phone calls from a physiotherapist (PT) for the first 8 weeks  Continued use of Fitbit and FitViz independently up to week 12  **Type of activity tracker:** Fitbit  **Goal:** Increase physical activity and manage joint symptoms  **Intervention duration (weeks):** 12 | Waitlist control | **Physical activity:** Steps/day, measured with a Fitbit |  |
| 29. | *Li et al.* 2023  (China) | **N:** 35  **Mean age (SD) control group:** 68.34 (5.13)  **Mean age (SD) intervention group:** 68.59 (3.88)  **Inclusion criteria:**  *-Age:* > 60 yrs  *-Health Condition:* Chronic lifestyle diseases (DM, CVDs etc)  **-***Source of recruitment:* Community  -*Mobility status:* not reported  -*Physical activity status:* physically inactive, <600 metabolic equivalent (METs) min/week calculated from the International Physical Activity Questionnaire (IPAQ) (long form) | **Intervention:** aerobic exercise intervention based on the capacity, opportunity, motivation-behaviour (COM-B) model on physical activity and health-related outcomes.  **Type of activity tracker:** a wearable smart sports bracelet (embedded with a PPG Biosensor Smart 4e sports bracelet [model C6] and an app [Day Band])  **Goal:** participant generated goals  **Intervention duration (weeks)***:*12 | Education booklet and WeChat platform with weekly updates about relevant PA | **Physical activity:** Steps/day measured with a wearable smart sports bracelet |  |
| 30. | *Lynch et al.* 2019  (Canada) | **N:** 83 (women)  **Mean age (SD) control group:** 61.9 (7.0)  **Mean age (SD) intervention group:** 61.3 (5.9)  **Inclusion criteria:**  *-Age:* > 50  *-Health Condition:* breast cancer  **-***Source of recruitment:* Register for and the Breast Cancer Network of Australia’s Review and Survey Group, 2 national registers of volunteers, Community and inactive retirees  -*Mobility status:* not reported  -*Physical activity status:* fewer than 75 minutes of MVPA per week and more than 7 hours of sedentary behavior per day. | **Intervention:**  Behavioural feedback and goal-setting sessions, Wearable technology activity monitor, Telephone-delivered behavioural counselling  **Type of activity tracker:** Garmin Vivofit 2  **Goal:** participant generated goals  **Intervention duration (weeks)***:*12 | Delayed intervention | **Physical activity:** Steps/day measured with a Garmin Vivofit 2 |  |
| 31. | *Mc Mohan et al. 2024*  *(USA)* | **N:** 309  Mean age (SD):  **Inclusion criteria:**  -*Age:* ≥70 years  *-Health Condition*: chronic conditions  *-Source of recruitment*: Community-dwelling  -*Mobility status*: NA  -*Physical activity status*: not meeting the national guidelines  recommended by the Physical Activity Guidelines for Americans Advisory Committee | **Intervention:** Behaviour Change Strategies (Intrapersonal Strategies +Interpersonal Strategies); activity monitor; Otago exercise program  **Type of activity tracker:** wearable Physical Activity monitor (PAM)  **Goal:** Patient-tailored goals  **Intervention duration (weeks):**1 | Attention control | **Physical activity:** steps/day measured with a Physical Activity monitor (PAM)- type of PAM not specified |  |
| 32. | *Mendoza et al.* 2015  (Chile) | **N:** 102  **Mean age (SD) control group:** 68 (8)  **Mean age (SD) intervention group:** 70 (10)  **Inclusion criteria:**  -*Age*: ⩾40 years  -*Health Condition:* COPD  -*Source of recruitment:* Hospital  -*Mobility status:* Not reported  *-Physical activity status:* Not reported | **Intervention:** Monthly physical activity counselling; given a pedometer; daily steps diary  **Type of activity tracker:** Tanita PD724 Triaxial pedometer  **Goal:** Patients had to increase between 3000 to 9000 steps/day following a specific protocol based on their average step count for the preceding week  **Intervention duration (weeks):** 12 | Monthly physical activity counselling | **Physical activity:** Steps/day, measured with a pedometer (Tanita PD724). Pedometer placement was not reported. **Quality of life:** St. George's respiratory questionnaire |  |
| 33. | *Menkin et al.* 2019  (USA) | **N:** 233  **Mean age (SD) control group:** 73.6(0.6)  **Mean age (SD) intervention group:** 74.1(0.6)  **Inclusion criteria:**  *-Age: >60 yrs*  *-Health Condition:* Hypertension (risk of stroke)  **-***Source of recruitment:* community senior centers of Latino, Korean, Chinese & and black old adults’ hospital  -*Mobility status:* able to walk in 10m walk test  -*Physical activity status:* not reported | **Intervention:** AT-based feedback + behavioural change theory to motivate walking  **Type of activity tracker:** Fitbit Zip pedometer  **Goal:** personalized goal setting  **Intervention duration (weeks)***:* 4 | Waitlist control | **Physical activity:** Steps/day measured with a pedometer  **Quality of life:** Physical-health–related Quality of life (QOL)  **Mental health:** 9-item Patient Health Questionnaire |  |
| 34. | *Nagai et al.* 2018  (Japan) | **N:***41*  **Mean age (SD) control group:** 81.2(7.3)  **Mean age (SD) intervention group:** 81.8(7.0)  **Inclusion criteria:**  *-Age: >65 years*  *-Health Condition:* frailty  **-***Source of recruitment:* Community-dwelling  *Mobility status:* ability to walk independently (or using a cane)  -*Physical activity status:* not reported | **Intervention:** PA + Resistance training +AT  **Type of activity tracker:** accelerometer (ACTi band, TDKCo., Tokyo, Japan)  **Goal:** personalized goal setting  **Intervention duration (weeks)***:* 24 | Resistance training | **Physical activity:** Steps/day,  measured with an accelerometer  **Quality of life**: Health-related quality of life SF-8 physical component  **Mental Health**: 9-item Patient Health Questionnaire |  |
| 35. | *Nolan et.al.* 2017  (United Kingdom) | **N:** 122  **Mean age (SD) control group:** 68 (8)  **Mean age (SD) intervention group:** 69  (9)  **Inclusion criteria:**  *-Age:* ⩾35years  *-Health Condition:* COPD  **-***Source of recruitment:* Hospital  -*Mobility status:* Not reported  -*Physical activity status:* No  contraindications to exercise | **Intervention:**  Pulmonary rehabilitation; given a pedometer.  step-count diary  **Type of pedometer:** Yamax Digi-walker  CW700  **Goal:** daily pedometer step-count target (with  weekly review).  **Intervention duration (weeks)***:* 8 | Usual care (Pulmonary  rehabilitation) | **Physical activity:** Steps/day,  measured with a pedometer (Yamax  Digi-walker CW700) worn on waist  **Quality of life:** Health status  assessed with the Chronic  Respiratory Questionnaire (CRQ) |  |
| 36. | *Smith-Turchyn et al. 2024*  *(Canada)* | **N:** 18  Mean age (SD): 72 years (5.7)  **Inclusion criteria:**  -*Age*:>65 years  -*Health Condition*: Cancer  -*Source of recruitment:* community association resources  -*Mobility status*: NA  -*Physical activity status:* less than 150 min of  MVPA per week | **Intervention:** weekly 1-hour virtual sessions with a qualified exercise professional for 10 weeks**;** activity tracker  **Type of activity tracker:** Fitbit Inspire 2©  **Goal:** achieve exercise guidelines for older adults and cancer survivors  **Intervention duration (weeks):**10 | Another active intervention (exercise + activity tracker) | **Physical activity:** steps/day using Fitbit  **Mobility:** Six-minute walking distance (6MWD)  **Quality of life:** EuroQol Visual Analogue Scale (EQ-VAS score) |  |
| 37. | *Su et al. 2024*  *(Hong Kong)* | **N:** 43  Mean age (SD): Intervention group 63.38 ± 2.96  Control group 63.59 ± 3.74  **Inclusion criteria:**  -*Age*: ≥60 years  -*Health Condition*: coronary heart disease  -*Source of recruitment*: a tertiary hospital  -*Mobility status*:NA  -*Physical activity status:* no prescribed physical activity restriction | **Intervention:** individualized in-person assessment; e-coaching sessions; cardiac telerehabilitation (CTR)  **Type of activity tracker:** Pedometer (Mi band)  **Goal:** personal behavioural  change goals with action plans  **Intervention duration (weeks):**12 | Standard care | **Physical activity: steps/day**  **Quality of life:** Health-Related Quality of Life (HRQOL) by *MacNew Heart Disease*  **Mental health**:21-item Depression  Anxiety Stress Scale 21 (DASS-21) |  |
| 38. | *Talbot et al.* 2003  (USA) | **N:** 34  **Mean age (SD) control group:** 71 (5)  **Mean age (SD) intervention group:** 70 (7)  **Inclusion criteria:**  -*Age:* ⩾60years  *-Health Condition:* Osteoarthritis  -*Source of recruitment:* Community  -*Mobility status:* Not reported  -*Physical activity status:* No contraindications to exercise | **Intervention:** Given a pedometer; a daily steps diary; an educational physical activity booklet; arthritis self-management program  **Type of activity tracker:** Yamax Digi-walker SW200 **Goal:** Monthly increase of daily steps by 10%  **Intervention duration (weeks):** 12 | Education (arthritis self-management program) | **Physical activity:** Steps/day, measured with a pedometer (Yamax Digi-Walker SW-200) worn on waist  **Mobility:** Chair and stand test |  |
| 39. | *Tew et al.* 2015  (United Kingdom) | **N**:16  **Mean age (SD) control group:** 68 (14)  **Mean age (SD) intervention group:** 69 (8)  **Inclusion criteria**:  -Age: ⩾18 years  -*Health Condition:* Intermittent claudication  -*Source of recruitment:* Hospital  -*Mobility status:* No limited walking  -*Physical activity status:* No contraindications to exercise | **Intervention:** Given a pedometer, educational session promoting self-managed walking and self-regulatory skills such as goal setting  **Type of activity tracker:** Yamax Digi-Walker SW200 **Goal:** Gradual increase of daily steps to more than 7500. **Intervention duration (weeks):** 6 | Usual care (educational leaflet) | **Physical activity:** Steps/day, measured with an accelerometer (ActiGraph GT3X+). Accelerometer placement not reported  **Quality of life:** EQ-5D utility score |  |
| 40. | *Van Dyck et al.* 2013  (Belgium) | **N:** 92  **Mean age (SD):** 62 (9)  **Inclusion criteria:**  -*Age:* 35-75 years -*Health Condition*: Type 2 diabetes  -*Source of recruitment:* Hospital  -*Mobility status:* Not reported  -*Physical activity status:* No physical activity limitations | **Intervention:** Given a pedometer, a face-to- face physical activity counselling followed by telephone contacts, daily steps diary  **Type of activity tracker:** Omron M6  **Goal:** Gradual progression starting from patient’s baseline levels.  **Intervention duration (weeks):** 24 | No intervention | ***Physical activity:*** Steps/day, measured with a pedometer (Omron M6) worn on waist |  |
| 41. | *Varas et al.* 2018  (Spain) | **N:**40  **Mean age (SD) control group:** 64.8±9.1  **Mean age (SD) intervention group:** 69.5±7.4  **Inclusion criteria:**  *-Age: not given*  *-Health Condition:* COPD  **-***Source of recruitment: Hospital*  -*Mobility status:* able to walk  -*Physical activity status:* (less than 30 min of moderate exercise per day | **Intervention:** Pulmonary rehabilitation; Activity Tracker; goal setting  **Type of activity tracker:** pedometer  **Goal:** personalised goal setting  **Intervention duration (weeks)***:* 13 | Education +usual care | **Physical activity:** Steps/day,  measured with a pedometer |  |
| 42. | *Widyastuti et al.* 2018  (Indonesia) | **N:**40  **Mean age (SD) control group:** 61.2(6.7)  **Mean age (SD) intervention group:** 68.3(6.7)  **Inclusion criteria:**  *-Age: 45–75-year-olds*  *-Health Condition:* COPD  **-***Source of recruitment: Outpatient clinic*  -*Mobility status:* able to walk  -*Physical activity status:* not reported | **Intervention:** Home-based pedometer assisted group  **Type of activity tracker:** pedometer (OmronHJ321)  **Goal:** personalised goal setting  **Intervention duration (weeks)***:* 6 | Supervised exercise training program | **Physical activity:** Steps/day,  measured with a pedometer |  |
| 43. | *Wootton et al.* 2019  (Australia) | **N:** 86  **Mean age (SD) control group**: 69 (9)  **Mean age (SD) intervention group**: 70 (7)  **Inclusion criteria:**  -*Age*: Not reported  -*Health Condition:* COPD  -*Source of recruitment:* Outpatient  -*Mobility status:* Not reported  -*Physical activity status:* Not reported | **Intervention:** telephone calls, biofeedback provided via pedometer  **Type of activity tracker:** (G-Sensor accelerometer, Pedometers Australia, Cannington, WA, Australia)  **Goal:** Progressive goal setting (based on pedometer data)  **Intervention duration (weeks):** 52 | Usual care | **Physical activity:** Steps/day, measured with a pedometer Pedometer placement was not reported |  |
| 44. | *Yates et al.* 2009  (United Kingdom) | **N**: 53  **Mean age (SD) control group:** 65 (10)  **Mean age (SD) intervention group**: 66 (8)  **Inclusion criteria:**  -*Age:* Not reported  -*Health Condition:* Impaired glucose tolerance  -*Source of recruitment:* Primary care  -*Mobility status:* Not reported  -*Physical activity status:* Not reported | **Intervention:** A single-session group-based education program addressing impaired glucose tolerance and physical activity counselling; pedometer with step-based physical activity goals  **Type of activity tracker:** Yamax Digi-Walker SW200  **Goal:** Sedentary: Increase by 3000 steps/day; ⩾6000 steps/day: Reach 9000 steps/day; >9000 steps/day: Maintain or increase step  **Intervention duration (weeks):** 52 | Usual care (education) | **Physical activity:** Steps/day, measured with a pedometer (New Lifestyles NL-800). Pedometer placement was not reported |  |
