# Supplementary material for: Effectiveness of interventions using activity trackers and smartphone applications for increasing physical activity in older adults with health conditions: a systematic review with meta-analysis
Source: J Frailty Aging. 2026 Aug 31;15(4):100180. doi: 10.1016/j.tjfa.2026.100180 (PMC13558122; doi:10.1016/j.tjfa.2026.100180)
Supplement: Supplementary file 5 [file mmc5.docx]

**Figure 6 (a):** Forest plot comparing activity trackers versus minimal intervention for physical activity in different health conditions - subgroup analysis

**Figure 6 (b):** Forest plot comparing activity trackers versus minimal intervention for physical activity in different health conditions- sensitivity analysis

**Figure 7 (a):** Forest plot comparing activity trackers versus minimal intervention for mobility in different health conditions subgroup analysis

**Figure 7 (b):** Forest plot comparing activity trackers versus minimal intervention for mobility in different health conditions sensitivity analysis

**Figure 8 (a):** Forest plot comparing activity trackers versus minimal intervention for Quality of life in different health conditions subgroup analysis

**Figure 8 (b):** Forest plot comparing activity trackers versus minimal intervention for Quality of life in different health conditions sensitivity analysis

**Figure 9 (a):** Forest plot comparing activity trackers versus minimal intervention for Mental health in different health conditions subgroup analysis

**Figure 9 (b):** Forest plot comparing activity trackers versus minimal intervention for Mental health in different health conditions sensitivity analysis

| Table 4. Summary of the certainty of evidence and strength of recommendation (GRADE) for Subgroup analysis | | | | | | | | | |
| --- | --- | --- | --- | --- | --- | --- | --- | --- | --- |
| Quality Assessment | | | | | | | | | |
| Subgroup-analysis | **Indirectness^1^** | **Study limitations^2^** | | | **Inconsistency^3^** | **Imprecision^4^** | **Publication bias^5^** | **Overall** | |
| *Activity tracker vs Minimal intervention* | | | | | | | | | |
| *Physical activity* | | | | | | | | | |
| Cancer |  |  | | |  | ↓ |  | Moderate | |
| Chronic conditions |  |  | | | ↓↓ |  |  | Low | |
| COPD |  | ↓ | | | ↓↓ |  |  | Very low | |
| CVD |  | ↓↓ | | | ↓ |  |  | Very low | |
| DM |  | ↓ | | | ↓↓ |  |  | Very low | |
| MSK conditions |  |  | | |  | ↓ |  | Moderate | |
| Stroke |  |  | | | ↓ |  |  | Moderate | |
| Total |  | ↓ | | | ↓↓ |  |  | Very low | |
| *Mobility* | | | | | | | | | |
| Cancer |  |  | | |  | ↓ |  | Moderate | |
| Chronic conditions |  | ↓↓ | | | ↓↓ | ↓ |  | Very low | |
| COPD |  | ↓ | | |  | ↓ |  | Low | |
| CVD |  |  | | |  | ↓ |  | Moderate | |
| MSK conditions |  | ↓ | | |  | ↓ |  | Low | |
| Stroke |  |  | | | ↓ | ↓↓ |  | Very low | |
| *Quality of life* | | | | | | | | | |
| Cancer |  | ↓ | | |  | ↓↓ |  | Very low | |
| Chronic conditions |  |  | | |  | ↓ |  | Moderate | |
| COPD |  | ↓ | | | ↓ |  |  | Low | |
| CVD |  |  | | | ↓↓ |  |  | Low | |
| Stroke |  |  | | |  | ↓↓ |  | Low | |
| *Mental health* | | | | | | | | | |
| Cancer |  | | ↓ |  | | ↓ |  | | Low |
| Chronic conditions |  | |  |  | | ↓ |  | | Moderate |
| COPD |  | | ↓ |  | | ↓ |  | | Low |
| CVD |  | |  |  | |  |  | | High |
| Stroke |  | |  | ↓↓ | | ↓↓ |  | | Very low |
| Abbreviations:  ^1^ Indirectness no downgrade as sensitivity analysis results is concordant  ^2^ >25% of participants from studies with low methodologic quality: PEDro score <6  ^3^ Heterogeneity > 60%  ^4^ <400 participants across all studies & SMD =0.5; MD >10% on either side of scale  ^5^ Serious small study effects suggested by funnel plot. Publication bias was not assessed because of fewer studies except in the comparison of physical activity vs minimal interventions  ↓ Downgraded | | | | | | | | | |

**Table 5.** Comparative effectiveness of Activity Trackers intervention vs minimal intervention on Physical activity, Mobility, Quality of life and Mental health.

| Condition | Comparison | Time Point | Effect Size [95% CI] | No. of Trials | Certainty of evidence |
| --- | --- | --- | --- | --- | --- |
| Physical activity | | | | | |
| Cancer | Minimal intervention | Short term | MD=**1153.05**,95%CI 199.85 to 2106.24 | 3 trials | Moderate |
| Chronic conditions | Minimal intervention | Short term | MD=**1159.07**,95% CI 388.29 to 1929.86 | 8 trials | Low |
| COPD | Minimal intervention | Short term | MD=**1245.12**,95% CI 428.22 to 2062.03 | 8 trials | Very low |
| CVD | Minimal intervention | Short term | MD=3377.59,95% CI ─ 135.36 to 6890.55 | 5 trials | Very low |
| DM | Minimal intervention | Short term | MD=**2014.09**,95% CI 824.92 to 3203.26 | 7 trials | Very low |
| MSK conditions | Minimal intervention | Short term | MD=881.75,95% CI ─ 56.34 to 1819.84 | 3 trials | Moderate |
| Stroke | Minimal intervention | Short term | MD=**1369.28**,95% CI 238.04 to 2500.51 | 3 trials | **Moderate** |
| Total | Minimal intervention | Short term | MD=1670.82,95% CI 1113.10 to 2228.53 | 37 trials | Very low |
| Mobility | | | | | |
| Cancer | Minimal intervention | Short term | SMD= ─ 0.38,95% CI ─ 1.04 to 0.27 | 1 trial | Moderate |
| Chronic conditions | Minimal intervention | Short term | SMD= ─ 0.01,95% CI ─ 1.24 to 1.23 | 3 trials | Very low |
| COPD | Minimal intervention | Short term | SMD= ─ 0.05,95% CI ─ 0.29 to 0.18 | 1 trial | Low |
| CVD | Minimal intervention | Short term | SMD= **0.22,**95% CI ─ 0.01 to 0.45 | 1 trial | **Moderate** |
| MSK conditions | Minimal intervention | Short term | SMD= ─ 0.27,95% CI ─ 0.95 to 0.40 | 1 trial | Low |
| Stroke | Minimal intervention | Short term | SMD= **0.64**,95% CI ─ 0.66 to 1.94 | 2 trials | Very low |
| Total | Minimal intervention | Short term | SMD= 1.01,95% CI ─ 0.25 to 2.28 | 9 trials | Very low |
| Quality of life | | | | | |
| Cancer | Minimal intervention | Short term | SMD= **0.58**, 95% CI ─ 0.05 to 1.22 | 1 trial | Very low |
| Chronic conditions | Minimal intervention | Short term | SMD= ─ 0.03, 95% CI ─ 0.24 to 0.17 | 4 trials | Moderate |
| COPD | Minimal intervention | Short term | SMD= ─ 0.12, 95% CI ─ 0.37 to 0.14 | 6 trials | Low |
| CVD | Minimal intervention | Short term | SMD= ─ 0.09, 95% CI ─ 1.68 to 1.50 | 4 trials | Low |
| Stroke | Minimal intervention | Short term | SMD= **0.93**, 95% CI ─ 0.42 to 2.27 | 1 trial | Low |
| Total | Minimal intervention | Short term | SMD= ─ 0.03, 95% CI ─ 0.43 to 0.37 | 16 trials | Low |
| Mental health | | | | | |
| Cancer | Minimal intervention | Short term | SMD= **0.39**,95% CI ─ 0.23 to 1.02 | 1 trial | Low |
| Chronic conditions | Minimal intervention | Short term | SMD = **0.26**,95% CI 0.03 to 0.49 | 2 trials | **Moderate** |
| COPD | Minimal intervention | Short term | SMD=0.00,95% CI ─ 0.23 to 0.23 | 1 trial | Low |
| CVD | Minimal intervention | Short term | SMD= ─ 0.08,95% CI ─ 0.25 to 0.08 | 3 trials | Moderate |
| Stroke | Minimal intervention | Short term | SMD= ─ 0.82,95% CI ─ 3.03 to 1.40 | 2 trials | Very low |
| Total | Minimal intervention | Short term | SMD=0.07,95% CI ─ 0.09 to 0.23 | 9 trials | **High** |

CI=Confidence interval; MD=Mean difference; SMD=Standardized mean difference’s; COPD=Chronic obstructive pulmonary disease; CVD=Cardiovascular disorders; DM=Diabetes milletus; MSK=Musculoskeletal
