# Supplementary material for: Effectiveness of interventions using activity trackers and smartphone applications for increasing physical activity in older adults with health conditions: a systematic review with meta-analysis
Source: J Frailty Aging. 2026 Aug 31;15(4):100180. doi: 10.1016/j.tjfa.2026.100180 (PMC13558122; doi:10.1016/j.tjfa.2026.100180)
Supplement: Supplementary file 6 [file mmc6.docx]

**Figure 2(a):** Forest plot comparing the effect of activity trackers versus minimal intervention on physical activity.

**Figure 2 (b):** Forest plot comparing activity trackers versus minimal intervention for short-term, intermediate, and long-term follow-up sensitivity analysis

**Figure 2 (c):** Funnel plot comparing activity trackers versus minimal intervention for Physical activity

**Figure 3**: Forest plot comparing the effect of activity trackers versus another active intervention on physical activity.

**Figure 4 (a):** Forest plot comparing activity trackers versus minimal intervention for mobility, quality of life and mental health

**Figure 4 (b):** Forest plot comparing activity trackers versus minimal intervention for mobility, quality of life and mental health sensitivity analysis

**Figure 4 (c):** Funnel Plot for Quality-of-life vs minimal intervention

**Figure 5:** Forest plot comparing activity trackers versus other active interventions for mobility, quality of life and mental health.

**Table 2.** Comparative effectiveness of Activity Trackers intervention vs Control on Physical activity, Mobility, Quality of life and Mental health.

| Comparison | Outcome | Time Point | Effect Size [95% CI] | No. of Trials | Certainty of evidence |
| --- | --- | --- | --- | --- | --- |
| Activity tracker vs Minimal intervention | *PA* | Nearest to intervention completion | MD = 1670.82,95% CI 1113.10 to 2228.53 | 37 trials | Very low |
|  | *PA* | Follow-up nearest to 6 months | MD = 1047.67, 95% CI ─ 962.50 to 3057.85 | 3 trials | Very low |
|  | *PA* | Follow-up nearest to 1 year | MD = 1837.84, 95% CI 979.76 to 2695.92 | 6 trials | Moderate |
|  | *PA* | Follow-up nearest to 2 years | MD = 315,95% CI ─ 1165.67 to 1795.67 | 1 trial | Low |
| Activity tracker vs other Active intervention | *PA* | Nearest to intervention completion | Not estimated | 3 trials | Very Low |
| Comparative effectiveness of Activity Trackers intervention vs Control on measures of Mobility, Quality of life, and Mental Health | | | | | |
| Activity tracker vs Minimal intervention | *Mobility*  Nearest to intervention completion | | SMD = 0.08, 95% CI ─ 0.27 to 0.42 | 9 trials | Very low |
| Activity tracker vs other Active intervention | *Mobility*  Nearest to intervention completion | | Not estimated | 2 trials | Very low |
| Activity tracker vs Minimal intervention | *Qol*  Nearest to intervention completion | | SMD = ─ 0.03, 95% CI ─ 0.43 to 0.37 | 16 trials | Low |
| Activity tracker vs other Active intervention | *Qol*  Nearest to intervention completion | | Not estimated | 3 trials | Very low |
| Activity tracker vs Minimal intervention | *Mental health*  Nearest to intervention completion | | SMD = 0.07, 95% CI ─ 0.09 to 0.23 | 9 trials | High |
| Activity tracker vs other Active intervention | *Mental health*  Nearest to intervention completion | | Not estimated | 1 trial | Very low |

Abbreviations: MD= Mean Difference; CI=Confidence Interval; SMD=Standardised mean difference; PA=Physical activity; Qol=Quality of life.

**Table 3.** Summary of the certainty of evidence and strength of recommendation (GRADE).

|  | Quality Assessment | | | | | |
| --- | --- | --- | --- | --- | --- | --- |
|  | **Downgraded** | | | | | **Overall** |
| Meta-analysis | **Indirectness**^1^ | **Study limitations**^2^ | **Inconsistency**^3^ | **Imprecision**^4^ | **Publication bias**^5^ |  |
| *Activity tracker vs Minimal intervention*  *Physical activity* | | | | | | |
| Short-term |  | ↓ | ↓↓ |  |  | Very low |
| Intermediate- Follow-up nearest to 6 months |  |  | ↓↓ | ↓ |  | Very low |
| Long-term Follow-up nearest to 1 year |  |  | ↓ |  |  | Moderate |
| Long-term Follow-up nearest to 2 years |  |  |  | ↓↓ |  | Low |
|  | Secondary outcomes | | | | | |
|  | *Mobility* | | | | | |
| Short-term |  |  | ↓↓ |  |  | Low |
|  | *Quality of life* | | | | | |
| Short-term |  |  | ↓↓ |  |  | Low |
|  | *Mental health* | | | | | |
| Short-term |  |  |  |  |  | High |
|  | *Activity tracker vs other active intervention* | | | | | |
|  | *Physical activity* | | | | | |
| Short-term |  | ↓↓ |  | ↓↓ |  | Very low |
|  | Secondary outcomes | | | | | |
|  | *Mobility* | | | | | |
| Short-term |  | ↓ |  | ↓↓ |  | Very low |
|  | *Quality of life* | | | | | |
| Short-term |  | ↓ | ↓↓ | ↓↓ |  | Very low |
|  | *Mental health* | | | | | |
| Short-term |  | ↓ |  | ↓↓ |  | Very low |
| ^1^ Indirectness no downgrade as sensitivity analysis results is concordant  ^2^ >25% of participants from studies with low methodologic quality: PEDro score <6  ^3^ Heterogeneity > 60%  ^4^ <400 participants across all studies & SMD =0.5; MD >10% on either side of scale  ^5^ Serious small study effects suggested by funnel plot. Publication bias was not assessed because of fewer studies except in the comparison of physical activity vs minimal interventions.  ↓ Downgraded | | | | | | |
